# Enantiodiscriminating Lipophilic Liquid Membrane-Based Assay for High-Throughput Nanomolar Enantioenrichment of Chiral Building Blocks

**DOI:** 10.3390/membranes13010094

**Published:** 2023-01-11

**Authors:** Bálint Jávor, Panna Vezse, Ádám Golcs, Péter Huszthy, Tünde Tóth

**Affiliations:** 1 Department of Organic Chemistry and Technology, Budapest University of Technology and Economics, Szent Gellért tér 4, H-1111 Budapest, Hungary; 2 Centre for Energy Research, Konkoly-Thege Miklós út 29-33, H-1121 Budapest, Hungary

**Keywords:** liquid membrane, optical resolution, enantiomeric recognition, passive diffusion, enantioenrichment

## Abstract

The reported optical resolution method was designed to support high-throughput enantioseparation of molecular building blocks obtained by automated small-scale synthetic methods. Lipophilic esters of common resolving agents were prepared and used as liquid membranes on the indifferent polymer surface of a microtiter assay. Chiral model compounds were enriched in one of the enantiomers starting from the aqueous solutions of their racemic mixture. Enantiodiscrimination was provided by forming diastereomeric coordination complexes of lipophilic enantiopure esters with the enantiomers of the chiral building blocks inside the liquid membranes. This enantiomeric recognition resulted in a greater distribution ratio of the preferred isomer in the membrane phase, thus the process enables a simultaneous enantioenrichment of the solutions outside the membrane. This paper reports a novel microplate-integrated stereoselective membrane enrichment technique satisfying the need for automatable enantioseparation on a subpreparative scale.

## 1. Introduction

Enantioseparation under the preparative scale means an increasingly relevant problem today since the state-of-the-art analytical methods require fewer amounts of enantiomerically pure compounds to be prepared. Supramolecular interactions enable separation even on a molecular scale if it is possible to trigger enantiomeric recognition toward a target substance. As a benefit of this phenomenon, tailorable applications can be developed with a high selectivity pre-determined for a particular substance or a group of structural analogues. In practice, the separation of nanomolar amounts of chiral molecules is performed most often by using chromatographic or electrophoretic methods based on chiral stationary phases [1,2,3]. These phases are typically immobilized host molecule-containing porous materials or molecular imprinted polymers [1,2,3]. The common drawbacks of these methods are the required great efforts for preliminary method development and the complicated and expensive preparation of the materials used for separation.

The need for separation of enantiomers from extremely small amounts of their mixtures often arises together with the need for high-throughput implementations. Although the most popular methods for high-throughput enantioseparation are liquid and supercritical chromatography and their coupled techniques [4,5,6,7], these methods mainly focus on sensing, i.e., determination of the enantiomeric excess, or profiling bioactive compounds [8,9,10,11,12,13] instead of separating them. On the other hand, the advent of nanotechnology [14,15] or microscale-device development opens great possibilities regarding the development of high-throughput separation processes [16].

Microscale systems containing selector molecules present promising potential based on their reduced reagent consumption, improved analysis speed, automated processing and high throughput [17]. There are various applicable nanomaterial-based strategies in this field. Microchip capillary electrophoresis has become a promising technique for enantioseparation due to the very short analysis time. Recently, the development of multichannel microchips has been among the most effective strategies, which can pave the way for generalizable, robust high-throughput enantioseparations [18,19,20,21,22]. This strategy can benefit from an enhanced specific surface area and the selective recognition of chiral selectors. As another alternative, graphene has a large surface area, good modifiability and desirable biocompatibility. Recently developed functionalized graphene oxide membranes tend to expand the field of high-throughput enantio- and bioseparations by tailorable microdevices [23,24,25].

Considering the mentioned drawbacks of the commonly used instrumental analytical methods, they can rarely be relevant alternatives for these purposes than membrane separations, which offer simpler, cheaper and more energy-efficient solutions [26,27]. Moreover, contrary to conventional separation methods, such as chromatography, crystallization and enzymatic kinetic resolution, membrane separations are continuous processes [28]. Nowadays, metal–organic frameworks (MOFs), covalent organic frameworks (COFs), zeolites or porous organic cages (POCs) can all potentially be used as chiral selector materials in advanced membrane matrixes [27]. Membrane techniques are particularly favorable when widely available simple-structure chiral selectors are able to induce enantiodiscrimination [25,29]. Moreover, in the case of liquid membranes, there is a good chance for integrability with microplate platforms as the bases of generally applied high-throughput applications. However, assay-based enantioselective applications mainly focus on determining the enantiomeric composition [30,31,32], while assay-based enantioseparation still remains an unexploited area of separation science.

**Figure 1:**
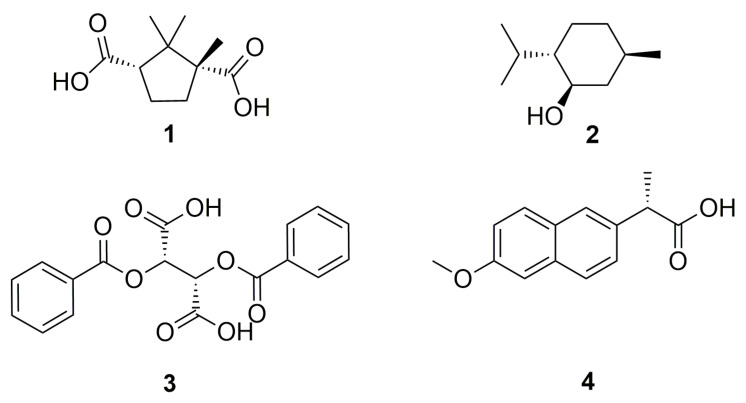
Enantiopure resolving agents as precursors of lipophilic enantioselector molecules used in liquid membranes.

Consequently, we aimed to prepare enantiodiscriminating liquid membranes, which can be suitable for the parallel separation of diverse chiral compounds when applying them on assay systems. The concentration gradient-governed membrane diffusion causes a greater distribution of the preferred enantiomer in the lipophilic layer, which causes enrichment of the less preferred one in the solution phase. To be able to separate isomers from generally applied aqueous solutions, highly apolar membranes are needed. According to our basic assumption, preparation of lipophilic derivatives of widely used, commercially available and relatively cheap enantiopure resolving agents leads to high tension viscous fluids as one-component membranes, which have enantiodiscriminating power. Among universally applicable simple resolving agents, derivatization of (+)-camphoric acid ((CA, (**1**)), (-)-menthol ((MEN, (**2**)), (-)-*O*,*O*′-dibenzoyl-L-tartaric acid ((DBTA, (**3**)) and (+)-naproxen ((NAP, (**4**)) was carried out by esterifications as cost-efficient one-step synthetic modifications, which typically result in minimal byproducts (Figure 1).

These precursors (**1**–**4** in Figure 1) of the new lipophilic resolving agents have already been used as excipients in various enantioselective applications for a long time.

In the case of CA (**1**) esters, only derivatives containing shorter alkyl chains have been characterized. These derivatives were primarily used as ligands in enantioselective syntheses and asymmetric inductions [33,34,35,36,37].

Among MEN (**2**) esters, derivatives containing a hexyl or a decyl chain had already been synthesized [38,39]. Furthermore, numerous other lipophilic analogues had also been prepared by the acylation with chlorinated fatty acids [39,40]. However, enzymatic synthesis methods are also applicable [41]. These esters have a broad application prospect as they can function as penetration promoters, can be applied in patches, cataplasms, ointments, gels, sprays and external liniments, as well as increase transcutaneous absorption of drug enantiomers [39,40].

DBTA (**3**) is intensively used for optical resolution based on the formation of diastereomeric complexes with various chiral compounds of stereoelectronically complementary structures. It tends to form hydrogen-bonded supramolecular coordination complexes with many chiral alcohols without basic groups. In this case, the guest molecule typically contains a proton-donating group and a fitting aliphatic chain or cycloalkane ring [42,43]. Not only alcohols but also amino acids [44], chiral *P*-ligands [45] or even atropisomers [46] can stereoselectively be bonded by this inexpensive and readily available resolving agent. These classical coordination-mediated resolutions can be performed even on a scale of kilograms. During the synthesis of DBTA esters, the introduction of the aromatic units is typically carried out in the last step by acylation with an acid chloride, after the esterification has already been performed. The bis (hexyl ester) derivative of DBTA was previously prepared according to a similar procedure to provide a renewable plasticizer [47]. Other analogues with longer alkyl chains have not been reported yet. However, their modified derivatives can also be successfully used for the preparation of enantioselective MOFs [48] or chiral coordination polymers [49].

NAP (**4**) has long been known as a non-steroidal anti-inflammatory drug. The acid function and the crystallization tendency of NAP and its derivatives can efficiently be exploited for the optical resolution of amines by diastereomeric salt formation [50,51]. Its lipophilic derivatives can be used as pro-drugs with enhanced absorption [52].

Conventional esterifications are equilibrium-limited homogeneous catalytic reactions, thus the catalysts (typically H_2_SO_4_, HCl or H_3_PO_4_) cannot be easily removed. Thus, heterogeneous catalysis becomes a more attractive alternative. Both ion-exchange resin and molecular sieves can be useful materials of improved methods. The former can be applied for the replacement of homogeneous acid catalysts [53], the latter is usually used in enzyme-catalyzed syntheses for shifting the equilibrium of the process [54]. By the combination of these additives, we report here a new heterogeneous catalytic method to replace direct esterifications for providing the desired lipophilic selector molecules in a more efficient and simplified way.

The proposed enantiopure lipophilic esters can be applied as chiral selectors or enantiodiscriminating liquid membranes in multiwell-assay systems for high-throughput purification of a great variety of chiral organic compounds. An investigation of the applicability along with future perspectives are also reported.

## 2. Experimental Section

### 2.1. Materials and Apparatus

Starting materials and reagents were purchased from Sigma-Aldrich Corporation (USA, owned by Merck, Darmstadt, Germany) and used without purification unless otherwise noted. Amberlite^®^ IR-120 acidic ion-exchange resin (Merck, Darmstadt, Germany) and 4 Å molecular sieve (1.6–2.6 mm beads, sodium aluminosilicate; Merck, Darmstadt, Germany) were used for heterogeneous catalytic esterifications. Solvents were dried and purified according to well-established methods [55]. Silica Gel 60 F254 (Merck, Darmstadt, Germany) plates were used for thin-layer chromatography (TLC). All reactions were monitored by TLC and visualized by UV lamp or by using phosphomolybdic acid (5% in ethanol) as a stain. Ratios of solvents for the eluents are given in volumes (mL/mL). Evaporations were carried out under reduced pressure unless otherwise stated. Sterile 96-well MultiScreen_HTS_ IP filter plates (indifferent PVDF membrane with 0.45 μm pore size, polyacrylate body, 0.28 cm^2^ flat-bottom wells; Merck, Darmstadt, Germany) were used for enantioseparation experiments. The acceptor site of the filter was covered with a homemade polyacrylate sheet to inhibit the material passage through the filter.

The new compounds were characterized by their physical constants such as melting point, thin-layer chromatography retention factor (*R*_f_), IR, ^1^H-NMR and ^13^C-NMR spectroscopies and HRMS spectrometry. Melting points were obtained on a Boetius micro-melting point apparatus and are uncorrected. Infrared spectra were recorded on a Bruker Alpha-T FT-IR spectrometer (Bruker Corporation, Billerica, MA, USA) using KBr pastilles. ^1^H- (300 MHz) and ^13^C- (75 MHz) NMR spectra were recorded on a Bruker 300 Avance spectrometer (Bruker Corporation, Billerica, MA, USA). ^1^H- (500 MHz) and ^13^C- (125 MHz) NMR spectra were obtained on a Bruker DRX-500 Avance spectrometer (Bruker Corporation, Billerica, MA, USA). HRMS analysis was carried out on a Thermo Velos Pro Orbitrap Elite (Thermo Fisher Scientific, Dreieich, Germany) system. The ionization method was ESI and was operated in positive ion mode. The protonated molecular ion peak was fragmented by CID at a normalized collision energy of 35–45%. The sample was dissolved in methanol. Data acquisition and analysis were accomplished with Xcalibur software version 2.2 (Thermo Fisher Scientific, Dreieich, Germany).

For incubating the assays, Boeco OS 20 digital orbital microtiter plate shaker (Boeckel + Co., Hamburg, Germany) was used.

UV–Vis spectra were recorded on a UNICAM UV4-100 spectrophotometer controlled by VIZION 3.4 software (ATI UNICAM, Knutsford, UK). Quartz cuvettes with a path length of 1 cm were used in all cases. OriginPro 8.6 (OriginLab Corp., Northampton, MA, USA) software was used for evaluating the spectrophotometric measurements. Optical rotations were obtained on a Perkin-Elmer 241 polarimeter (PerkinElmer Inc., Waltham, MA, USA), which was calibrated by measuring the optical rotations of both enantiomers of menthol.

### 2.2. Measurements

Selector molecules obtained as an oil (**13**, **14**, **17** and **18**, see Section 3.1) were directly used as lipophilic liquid membranes, while selector molecules obtained as a solid (**8**–**10**, **15** and **16**, see Section 3.1) were applied in dissolved form on the surface of the PVDF filter of the microtiter plate. In the former case, 7.5 × 10^−6^ mol·L^−1^ selector molecule in 10 μL hexane was evenly spread on the bottom of each well. The assay was heated with an infrared lamp at 40 °C for 8 h to evaporate the hexane. In the latter case, the same amount of selector molecule was dissolved in 10 μL decan-1-ol (melting point: 6–7 °C) and this solution was applied as liquid membrane in each well. Enantioseparation experiments were carried out starting from the 5.0 × 10^−2^ mol·L^−1^, pH = 1.0 aqueous solutions of the chiral model compounds. From these solutions, 150 μL was added to each well using a multichannel pipette. During the initial 5 min of the 120-min incubation, the microtiter plate was bidirectionally shaken at 100 rpm using a microplate shaker. The enantioseparation process was characterized by the distribution ratio (*D*) and the selectivity factor (*β*) using the following equations:

(1) $$D_{E,aq}=\frac{ratio\;of\;E\;in\;the\;aqueous\;phase\;\left(\%\right)}{ratio\;of\;E\;in\;the\;lipophilic\;liquid\;membrane\;\left(\%\right)}$$

(2) $$\beta =\frac{D_{E,l,aq}}{D_{E,p,aq}}$$

where *D_E*,*aq_* is the distribution ratio of a single enantiomer in the aqueous phase, while *β* is the proportion of the distribution ratio of the less preferred enantiomer (*D_E*,*l*,*aq_*) and that of the preferred one (*D_E,p,aq_*) in the aqueous phase.

The distribution ratio between the lipophilic liquid membrane and the aqueous solution phase was determined based on UV–Vis absorption measurements. Samples from the aqueous phase (10 μL) were 500-fold diluted with aqueous HCl solution (pH = 1.0). Concentrations of the model compounds in these samples were determined by 4-point UV–Vis calibration curves (10^−4^–10^−5^ mol·L^−1^), which can be found in the Supporting Information.

In the case of preliminary studies on separability, the expected ratio of enantiomeric enrichment was estimated based on the kinetic studies on the isolated single enantiomers. The estimated *ee*% values were calculated according to the following equation:

(3) $$ee_{aq}\left(\%\right)=\frac{R_{E,l,aq.}-R_{E,p,aq.}}{R_{E,l,aq.}+R_{E,p,aq.}}\cdot 100$$

where *R_E*,*l*,*aq._* is the percentage ratio of the less preferred enantiomer in the aqueous phase, while *R_E,p,aq._* is that for the preferred one.

In the case of separation experiments starting from the racemic mixtures of the enantiomers, the efficiency of the process was characterized by the estimated enantiomeric excess (*ee*%) of the less preferred enantiomer in the aqueous phase. The *ee*% values were estimated by the optical purity based on optical rotation measurements. The optical purity data were determined by measuring the specific rotations of the samples [*α*] comparing them to the optical rotation of the enantiomerically pure component [*α_max_*] assuming a linear correlation (from −152 to +152° for **19**, from −20 to +20° for **20**, from −46 to +46° for **21**, from −37 to +37° for **22**, from −151 to +151° for **23**; for formulas see Section 3.2). (The errors of the optical purity values were less than ±2.0%. However, the *Horeau*-effect can enlarge the deviation of *ee*% estimations, especially around 50 *ee*%). Optical rotation measurements were carried out at concentration c = 0.4 g/100 mL using a Hg (578 nm) linearly polarized light source in aqueous HCl solution (pH = 1.0). During the sample preparation 100–100 μL aqueous solutions were collected from 20 parallel separations, then combined samples were evaporated and the solid residue was dissolved in 500 μL aqueous HCl solution (pH = 1.0).

All of the experiments were carried out at 15 °C. Each reported result was determined as the average of at least 3 independent measurements. The separability of the enantiomers was validated by statistical evaluation methods. The significance of the differences between the averages of obtained data was investigated by two-sample *t*-tests at a significance level of 95% (the equality of the standard deviations was checked by *F*-probe, the standard deviations were calculated from 4 parallel measurements, thus degrees of freedom = 6). If the value of our calculated statistic exceeded the corresponding *t* parameter (|*t*| ≥ *t_Student_*), the null hypothesis was rejected, meaning that there is a significant difference between the distribution of the enantiomers, thus enantioseparation was performed.

### 2.3. Synthesis of the Lipophilic Selector Molecules (see Scheme 1)

#### 2.3.1. General Method for Acid-Catalyzed Direct Esterification

A mixture of acid (**1** or **3** or **4**) (1.0 molar eq.), alcohol (**5** or **6** or **7**) (150 molar eq.) and catalytic amount (0.1 molar eq.) of 98% H_2_SO_4_ was stirred at 80 °C for 24 h. The mixture was taken up in water (200 mL/1 g acid). The phases were shaken well and separated. The pH of the aqueous phase was adjusted to 10 with saturated aqueous Na_2_CO_3_ solution, then the aqueous phase was extracted with ethyl acetate (3 × 200 mL/1 g acid). The combined organic phase was shaken with saturated brine (100 mL/1 g acid), dried over MgSO_4_, filtered and the solvent was removed. The crude products were purified by column chromatography on silica gel adsorbent using hexane:ethyl acetate 10:1 as an eluent to give the title compounds (**8**–**10**, **15**–**18**).

#### 2.3.2. General Method for Esterification by Acid Chlorides

Thionyl chloride (1.5 molar eq.) and catalytic amount of DMF (0.1 molar eq. to form *Vilsmeier*-adduct) were slowly added to a stirred solution of acid (1.0 molar eq.) (**1** or **3** or **4** or **11** or **12**) in dry and pure dichloroethane (40 mL/1 g acid) at 0 °C. The mixture was stirred at 0 °C for 15 min and then at room temperature for 4 h. Thionyl chloride was carefully removed by distillation while taking care not to evaporate the volatile components making the acid chloride dry. If necessary, the distillation should be carried out several times by maintaining a small amount of dichloroethane. After evaporating the most of reagent, alcohols (**2** or **5**–**7**) (1.5 molar eq.) were added dropwise to the solution of acid chloride in dichloroethane at 0 °C, then TEA (5.0 molar eq.) was added. The temperature of the reaction mixture was raised to 50 °C and the mixture was stirred at this temperature for 12 h under Ar. Then the mixture was taken up in ice-cold water (100 mL/1 g acid). The phases were shaken well and separated. Extraction was carried out with dichloroethane (3 × 100 mL/1 g acid) upon adjusting the pH of the aqueous phase to **3** (to remove TEA*HCl) and subsequently to **10** (to remove the remaining amount of acid) by using 5% aqueous solution of HCl and saturated aqueous Na_2_CO_3_ solution, respectively. The combined organic phase was shaken with saturated brine (100 mL/1 g acid), dried over MgSO_4_, filtered and the solvent was removed. The crude products were purified by column chromatography on silica gel using hexane:ethyl acetate 10:1 as an eluent to give the title compounds (**8**–**10**, **13**–**18**).

#### 2.3.3. Novel Heterogeneous Catalytic Synthesis Method for Direct Esterification

Lipophilic alcohol (**5**–**7**) (150 molar eq.) was added to the stirred mixture of enantiopure acid (1.0 molar eq.), 4 Å molecular sieve (2.0 mass eq.) and acidic ion-exchange resign (1.0 mass eq.) at room temperature. The heterogenous mixture was vigorously stirred at 80 °C for 12 h. The solid materials were removed by suction filtration using a glass filter of 10–16 μm-pore size. The excess of alcohols (**5**–**7**) was removed by distillation under reduced pressure to provide the title compounds (**8**–**10**, **15**–**17**).

### 2.4. Characterization of the Applied Selector Molecules

Among the applied synthetic procedures, only the most effective one was highlighted for each target compound. Further discussion can be found in Section 3.1. NMR spectra of the new compounds can be found in the Supporting Information.

#### 2.4.1. 1,3-Dihexyl-(1*R*,3*S*)-1,2,2-trimethylcyclopentane-1,3-dicarboxylate (CA-6, **8** in Scheme 1)

CA-6 (**8**) was synthesized according to the general procedure described in Section 2.3.3 starting from CA (**1**) (500 mg, 2.50 mmol) to provide the title compound (**8**) as a white solid (893 mg, 97%).

M.p. = 160 °C. *R*_f_ = 0.55 (silica gel TLC, hexane:ethyl acetate 4:1). ${\left[\alpha \right]}_{Hg\left(578\right)}^{15}$ = + 19 (c = 1.0, ethanol:water 95:5). ^1^H-NMR (500 MHz, CDCl_3_): *δ* [ppm]: 3.66 (t, *J* = 6.7 Hz, 4H), 2.92–2.80 (m, 1H), 2.64–2.49 (m, 1H), 2.27–2.13 (m, 1H), 1.95–1.79 (m, 1H), 1.60–1.57 (m, 8H), 1.40–1.34 (m, 12H), 0.91 (s, 12H). ^13^C-NMR (75 MHz, acetone-*d*_6_): *δ* [ppm]: 176.3, 174.4, 61.5, 55.8, 52.2, 45.7, 32.8, 32.7, 32.4, 31.8, 31.7, 31.6, 29.7, 29.5, 29.4, 29.2, 29.0, 28.7, 28.5, 28.2, 25.8, 25.5, 22.5, 22.3, 21.1, 20.7, 13.5. IR: *ν_max_* [cm^−1^]: 2924, 2855, 1736, 1695, 1458, 1376, 1345, 1273, 1235, 1167, 1124, 1056, 929, 721, 627, 550, 459. HRMS: *m*/*z* = [MH^+^]: 369.2929 (Calcd. for C_22_H_40_O_4_, 368.2927).

#### 2.4.2. 1,3-Dioctyl-(1*R*,3*S*)-1,2,2-trimethylcyclopentane-1,3-dicarboxylate (CA-8, **9** in Scheme 1)

CA-8 (**9**) was synthesized according to the general procedure described in Section 2.3.3 starting from CA (**1**) (500 mg, 2.50 mmol) to provide the title compound (**9**) as a white solid (1008 mg, 95%).

M.p. = 122 °C. *R*_f_ = 0.58 (silica gel TLC, hexane:ethyl acetate 4:1). ${\left[\alpha \right]}_{Hg\left(578\right)}^{15}$ = +13 (c = 1.0, ethanol:water 95:5). ^1^H-NMR (500 MHz, acetone-*d*_6_): *δ* [ppm]: 4.32 (t, *J* = 5.7 Hz, 4H), 2.44–2.36 (m, 4H), 2.16–2.14 (m, 2H), 2.04–1.97 (m, 4H), 1.73–1.65 (m, 4H), 1.49–1.46 (m, 2H), 1.25–1.22 (m, 16H), 0.78–0.75 (m, 12H). ^13^C-NMR (125 MHz, acetone-*d*_6_): *δ* [ppm]: 176.1, 174.2, 65.0, 61.5, 55.8, 52.1, 33.4, 32.9, 32.4, 31.7, 31.7, 29.9, 29.4, 26.2, 25.8, 24.7, 22.5, 22.3, 21.1, 20.7, 13.5. IR: *ν_max_* [cm^−1^]: 2954, 2923, 2854, 1731, 1456, 1369, 1245, 1173, 1106, 985, 722, 595, 458. HRMS: *m*/*z* = [MH^+^]: 425.3550 (Calcd. for C_26_H_48_O_4_, 424.3553).

#### 2.4.3. 1,3-Didodecyl-(1*R*,3*S*)-1,2,2-trimethylcyclopentane-1,3-dicarboxylate (CA-12, **10** in Scheme 1)

CA-12 (**10**) was synthesized according to the general procedure described in Section 2.3.3 starting from CA (**1**) (500 mg, 2.50 mmol) to provide the title compound (**10**) as a white solid (1287 mg, 96%).

M.p. = 95 °C. *R*_f_ = 0.60 (silica gel TLC, hexane:ethyl acetate 4:1). ${\left[\alpha \right]}_{Hg\left(578\right)}^{15}$ = +11 (c = 1.0, ethanol:water 95:5). ^1^H-NMR (500 MHz, CDCl_3_): *δ* [ppm]: 3.61 (t, *J* = 6.7 Hz, 4H), 2.64–2.52 (m, 1H), 2.41–2.09 (m, 8H), 2.04–1.86 (m, 3H), 1.82–1.73 (m, 4H), 1.69–1.55 (m, 1H), 1.54–1.31 (m, 5H), 1.31–1.00 (m, 28H), 0.99–0.90 (m, 4H), 0.90–0.87 (m, 6H). ^13^C-NMR (125 MHz, CDCl_3_): *δ* [ppm]: 172.7, 170.0, 54.4, 53.8, 45.3, 43.7, 37.3, 33.5, 32.3, 29.7, 24.5, 21.1, 20.8, 20.2, 14.2. IR: *ν_max_* [cm^−1^]: 2926, 2857, 1735, 1694, 1457, 1372, 1343, 1236, 1167, 1056, 927, 720, 627, 551, 460. HRMS: *m*/*z* = [MH^+^]: 537.4800 (Calcd. for C_34_H_64_O_4_, 536.4805).

#### 2.4.4. (1*R*,2*S*,5*R*)-5-Methyl-2-(propan-2-yl)cyclohexyl hexanoate (MEN-6, **13** in Scheme 1)

MEN-6 (**13**) was synthesized according to the general procedure described in Section 2.3.2 starting from MEN (**2**) (500 mg, 3.20 mmol) to provide the title compound (**13**) as a colorless oil (374 mg, 46%).

Physical and spectroscopic data concurred with those previously reported [38].

#### 2.4.5. (1*R*,2*S*,5*R*)-5-Methyl-2-(propan-2-yl)cyclohexyl decanoate (MEN-10, **14** in Scheme 1)

MEN-10 (**14**) was synthesized according to the general procedure described in Section 2.3.2 starting from MEN (**2**) (500 mg, 3.20 mmol) to provide the title compound (**14**) as a colorless oil (378 mg, 38%).

Physical and spectroscopic data concurred with those previously reported [39].

#### 2.4.6. 1,4-Dihexyl-(2*S*,3*S*)-2,3-bis(benzoyloxy)butanedioate (DBTA-6, **15** in Scheme 1)

DBTA-6 (**15**) was synthesized according to the general procedure described in Section 2.3.2 starting from DBTA (**3**) (500 mg, 1.40 mmol) to provide the title compound (**15**) as a white solid (612 mg, 83%).

Physical and spectroscopic data concurred with those previously reported [47].

#### 2.4.7. 1,4-Dioctyl-(2*S*,3*S*)-2,3-bis(benzoyloxy)butanedioate (DBTA-8, **16** in Scheme 1)

DBTA-8 (**16**) was synthesized according to the general procedure described in Section 2.3.2 starting from DBTA (**3**) (500 mg, 1.40 mmol) to provide the title compound (**16**) as a white solid (578 mg, 71%).

M.p. = 40 °C. *R*_f_ = 0.45 (silica gel TLC, hexane:ethyl acetate 4:1). ${\left[\alpha \right]}_{Hg\left(578\right)}^{15}$ = −106 (c = 1.0, ethanol:water 95:5). ^1^H-NMR (500 MHz, acetone-*d*_6_): *δ* [ppm]: 8.00–7.97 (m, 2H), 7.93–7.90 (m, 2H), 7.61–7.57 (m, 1H), 7.50–7.44 (m, 3H), 7.40–7.37 (m, 2H), 6.16–6.10 (m, 1H), 5.87–5.82 (m, 1H), 4.32 (t, *J* = 5.7 Hz, 2H), 3.92–3.90 (m, 2H), 1.80–1.72 (m, 2H), 1.70–1.58 (m, 2H), 1.44–1.39 (m, 20H), 0.73–0.69 (m, 6H). ^13^C-NMR (125 MHz, CDCl_3_): *δ* [ppm]: 166.8, 165.5, 133.6, 133.6, 130.1, 128.5, 70.9, 66.4, 37.0, 36.3, 31.9, 31.3, 29.7, 29.4, 28.4, 25.4, 22.7, 22.4, 14.1, 13.9. IR: *ν_max_* [cm^−1^]: 2958, 2931, 2858, 1766, 1730, 1602, 1450, 1415, 1314, 1243, 1105, 1069, 708, 502. HRMS: *m*/*z* = [MH^+^]: 583.3196 (Calcd. for C_34_H_46_O_8_, 582.3193).

#### 2.4.8. 1,4-Didodecyl(2*S*,3*S*)-2,3-bis(benzoyloxy)butanedioate (DBTA-12, **17** in Scheme 1)

DBTA-12 (**17**) was synthesized according to the general procedure described in Section 2.3.2 starting from DBTA (**3**) (500 mg, 1.40 mmol) to provide the title compound (**17**) as a colorless oil (632 mg, 65%).

*R*_f_ = 0.50 (silica gel TLC, hexane:ethyl acetate 4:1). ${\left[\alpha \right]}_{Hg\left(578\right)}^{15}$ = −124 (c = 2.0, ethanol:water 95:5). ^1^H-NMR (300 MHz, CDCl_3_): *δ* [ppm]: 8.03–7.97 (m, 10H), 7.40–7.37 (m, 1H), 7.29–7.27 (m, 1H), 4.15–4.09 (m, 2H), 3.65–3.48 (m, 2H), 1.69–1.60 (m, 1H), 1.60–1.49 (m, 1H), 1.45–1.35 (m, 2H), 1.34–1.14 (m, 36H), 0.86 (t, *J* = 6.4 Hz, 6H). ^13^C-NMR (75 MHz, CDCl_3_): *δ* [ppm]: 162.6, 161.2, 129.5, 128.4, 127.0, 64.1, 60.4, 45.3, 37.3, 36.5, 32.8, 31.9, 31.5, 29.6, 28.5, 25.8, 25.7, 22.7, 21.0, 14.1. IR: *ν_max_* [cm^−1^]: 2959, 2924, 2856, 1724, 1602, 1452, 1416, 1315, 1263, 1244, 1109, 1069, 709, 502. HRMS: *m*/*z* = [MH^+^]: 695.4450 (Calcd. for C_42_H_62_O_8_, 694.4445).

#### 2.4.9. Hexyl-(2*S*)-2-(6-methoxynaphthalen-2-yl)propanoate (NAP-6, **18** in Scheme 1)

NAP-6 (**18**) was synthesized according to the general procedure described in Section 2.3.2. starting from NAP (**4**) (500 mg, 2.17 mmol) to provide the title compound (**18**) as a colorless oil (477 mg, 70%).

${\left[\alpha \right]}_{Hg\left(578\right)}^{15}$ = +150 (c = 2.0, ethanol:water 95:5). Other physical and spectroscopic data concurred with those previously reported [56].

## 3. Results and Discussion

### 3.1. Synthesis of Enantiopure Lipophilic Esters Used as Chiral Selectors of Apolar Liquid Membranes

**Scheme 1:**
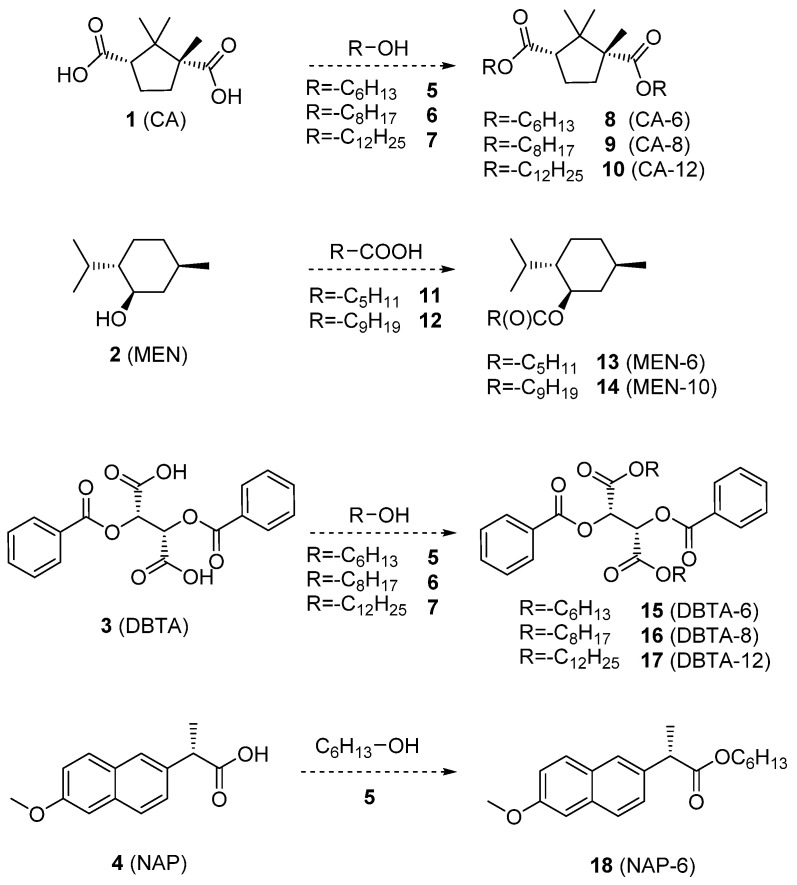
Synthetic modifications of the enantiopure resolving agents (**1**–**4**) to enhance their lipophilic character and provide the stability of the apolar liquid membranes for the enantioenrichment process.

As feasibility and availability were the key aspects of conceptualization, widely used and relatively cheap enantiopure scaffolds were modified by one-step esterifications. Reactions were carried out according to three different procedures detailed in Section 2.3. In the case of bifunctional scaffolds (**1**,**3**), both carboxylic groups were esterified to cover all the polar protic units, which tend to coordinate both enantiomers of racemic compounds with hydrogen bonds and to form salts with organic bases inside the membrane. As the basic concept was integrating apolar liquid membranes in microtiter assays, immobilization of selector molecules was implemented by the enhancement of their lipophilic character by introducing long alkyl chains into the molecules without affecting the chiral centers. These structural modifications enable non-covalent binding of the selector molecules to the membranes, which prevents their leaching to the aqueous solutions of the model compounds. The schematics for the summary of the synthetic work can be seen in Scheme 1.

If the applied selector molecule is an oil, it can be used as a membrane without additional matrix components, but it also needs to be insoluble in water. To study the influence of the length of the introduced alkyl chains, a series of lipophilic analogues have been prepared.

In the case of CA (**1**), all the three reported methods were attempted to synthesize the desired lipophilic selector molecules (**8**–**10**). A large excess of lipophilic alcohols (**5**–**7**) was used in all these cases. According to the experience, the homogeneous acid-catalyzed direct esterification (Section 2.3.1) resulted only in a partial conversion, while the acylation by acid chlorides (Section 2.3.2) resulted in more byproducts. Thus, the proposed novel heterogeneous catalytic method (Section 2.3.3) —using 4 Å molecular sieve for water removal (and for shifting the equilibrium in favor of the product) and acidic ion-exchange resin as a catalyst-proved to be the most efficient procedure resulting in the highest yield and needed no chromatographic purification of the products.

As in the case of synthesizing MEN esters (**13** and **14**), the alcohol (MEN, **2**) was the enantiopure scaffold, which is a solid material, no excess of it was used. Due to the need of solvent, the method described in Section 2.3.3 was not attempted and the reaction with acid chlorides clearly seemed to be the best choice.

The acid-catalyzed methods (Section 2.3.1 and Section 2.3.3) were not suitable for esterifying DBTA (3), since the benzoyl groups were unstable in acidic conditions. In the case of the preferred method (Section 2.3.2), benzoyloxy units also showed a high tendency to cleavage when the acid chloride derivative of DBTA was evaporated to dryness before the addition of the alcohols (**5**–**7**) and TEA. This is why some amount of the solvent had to be left until the thionyl chloride had been totally removed from the reaction mixture.

NAP ester **18** was obtained with high yields by all of the applied methods.

### 3.2. Parameter Optimization and Kinetic Properties

**Figure 2:**
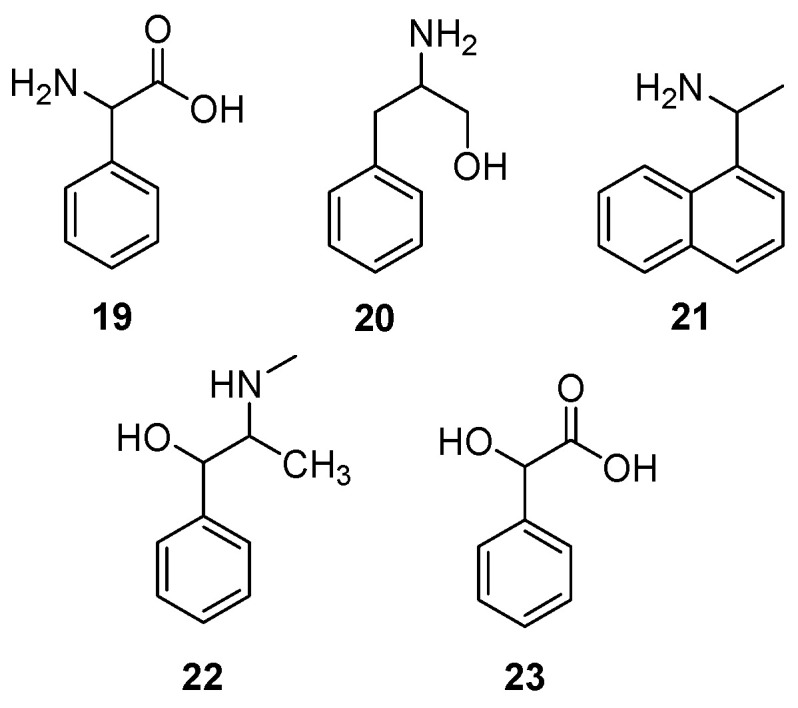
Schematics of model compounds used for enantioenrichment studies.

Enrichment of one of the enantiomers of chiral compounds was carried out using their aqueous solutions in favor of easier handling from the aspect of automation. Herein, we report a preliminary study on the extensibility of the procedure by using five chiral, small-molecule biogenic model compounds (**19**–**23** in Figure 2). These chiral building blocks were dissolved in aqueous HCl solutions (pH = 1.0) to increase solubility and to provide the same ionization state of the functional groups.

**Figure 3:**
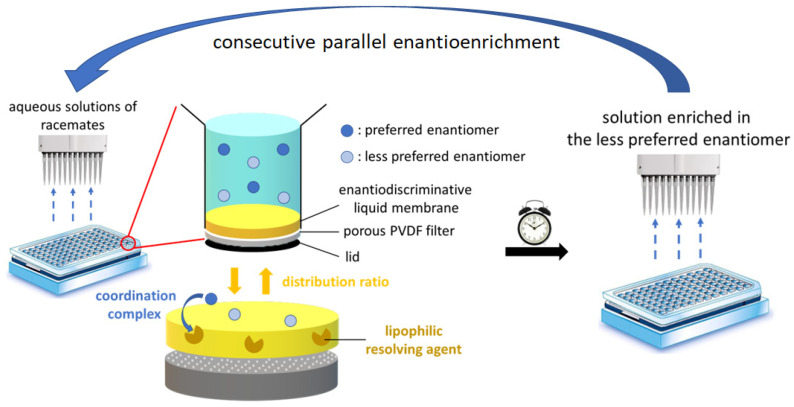
Concept of the novel high-throughput enantioenrichment method.

During the enrichment process, the solutions of the model compounds were added to each well of the assay. After contacting the surface of the lipophilic liquid membranes at the bottom of the wells, a distribution equilibrium begins to establish between the aqueous solution and the apolar bulk phase. By its nature, this distribution equilibrium is also strongly influenced by the polarity of the model compounds. On the other hand, thanks to the presence of the selector molecules, this polarity-based balance can be shifted by enantiomeric recognition. Under ideal conditions, the enantiomeric preference of the selector molecule can be significantly manifested. The selector molecules form coordination complexes of different stabilities with the enantiomers of the chiral model compounds inside the membrane phase, which results in a stereoselectivity of the membrane-affinity of the chiral agents. For comparability, the membranes contained an equimolar amount of selector molecules to chiral model compounds in all of the cases. After a proper incubation time, the aqueous phases can be enriched in the less favored enantiomer. Each separation step should be interrupted at an initial stage of the distribution equilibrium process, when enantiodiscriminative power is more strongly expressed compared to polarity-based interactions. Schematic representation of the process can be seen in Figure 3.

The initial goal of this preliminary study was to demonstrate that statistically significant enantiodiscrimination can take place by applying the proposed novel separation technique. Later, the separation efficiency can be increased by an extended parameter-optimization and separation can also be completed by subsequently repeating the initial membrane separation steps.

Unfortunately, several lipophilic esters of potentially applicable resolving agents can only be obtained as solid materials (**8**–**10**, **15**, **16**). In these cases, they needed to be dissolved to create liquid membranes with sufficient permeability for the dissolved chiral compounds. For eliminating the probability of non-stereoselective intermolecular interactions, the indifferent, apolar nature of the membrane matrix had to be maintained. As long-chain alkanes weakly dissolve the applied selector molecules, decan-1-ol was chosen as an alternative containing only one polar unit. For similar reasons, reducing the membrane thickness is favorable, and the content of selector molecules of the membranes should be maximized. Moreover, the volume of the membrane is required to be enough to evenly cover the surface of the bottom of the wells.

The relatively high melting points of the decan-1-ol and the enantiopure esters pointed the applicable minimal temperature to 15 °C. In order to achieve the best possible selectivity, studies were carried out at this temperature.

The most efficient separation could be reached by adjusting the ratio of the amount of the selector molecules to 50%, related to that of the racemic model compounds. In contrast, selector molecules were applied in an equimolar amount in the membranes for judging the statistical significance of enantiodiscrimination as a worst-case study (concentration values have only a secondary role besides the selector-guest ratio to accelerate the establishment of an equilibrium state by increasing the concentration gradient). These measurements were carried out by incubating the aqueous solutions of the single enantiomers separately using different membranes. The parallel monitoring of these aqueous phases led to investigating the timescale and the kinetic properties of the enantioenrichment process. Moreover, it gives a good estimation for the ideal incubation time, when the efficiency is optimal considering both the distribution ratio (*D*) and the enantioselectivity (*β*).

Initially, NAP-6 (**18**) was investigated as a single-component liquid membrane and also as a selector molecule in decane-1-ol to compare these two reported types of application. Studies on kinetic properties were carried out by recording the concentrations of the model compounds in the aqueous phase as a function of the incubation time. The diagrams obtained from the individual parallel studies on the pure enantiomers can be found in the Supporting Information. Differences between the concentrations at the same stage of the process indicate the expected rate of enantioenrichment. Experiments supported that the differences between the concentrations of opposite enantiomers continuously decreased during the progress of incubation. This is related to the fact that as time progresses, the polarity-based distribution characterizing the equilibrium state between the phases takes place gradually. At a later stage, this equilibrium process suppresses the differences in membrane affinities caused by the formation of coordination complexes of different stabilities. Consequently, separations should be interrupted at an early stage when enantiomeric discrimination is more strongly expressed. According to the expectations, the deviance of the measurements was lower in the case of determining larger concentrations. Considering both the required timescale for the establishment of the equilibrium distribution and the tendency of the distribution ratio (*D_E*,*aq._*)-changes, applicability was compared after 1 h of incubation time, conventionally. Separability of the enantiomers was characterized by the distribution ratios and their relative proportions defined as selectivity factor (*β*) (see Section 2.2). In all cases, *β* was assigned to the configuration favored by the selector. Table 1 shows the results from the individual parallel studies on the pure enantiomers of the model compounds (**19**–**23**).

It is clearly seen that the polarity-based distribution dominated more strongly when decane-1-ol was used as an additive. Due to the enlarged number of non-stereoselective interactions, separability decreased in comparison with NAP-6 (**18**) as a single-component liquid membrane. In addition, not only the possibility for non-stereoselective interactions but also the volume of the liquid membrane was increased in the former case while the amount of the applied selectors remained unchanged. It can also strengthen the affinity of the model compounds to diffuse into the lipophilic phase.

The determined concentrations succeeded the 50% ratio related to the selector molecules reflecting that the membranes can bind more guest molecules than it would follow from the expected 1:1 complex stoichiometry. It supported that the selectors coordinate both enantiomers but with different stabilities. Moreover, it can also be attributed to other possible stoichiometric ratios of coordination and also to the polarity-based affinities of the guest molecules to the organic phase. In summary, the equilibrium distribution of the guest molecules could only be weakly influenced by the additionally occurring enantiomeric recognition. However, by reducing the polarity-based effects to the minimal amount, the enantiodiscriminating power becomes exploitable according to the statistical evaluation of the data as the differences in *D_E*,*aq._*-values for opposite enantiomers are significant.

Surprisingly, the lipophilicity of the model compounds did not reflect from the results. It seemed, that the secondary binding forces have stronger effects on the membrane affinity than the expected solubility relations (log*D* at pH = 1.0 is: −2.1 for **19**, −2.3 for **20**, −0.5 for **21**, −1.9 for **22** and +0.9 for **23**, predicted by log*D* Predictor of ChemAxon, Budapest, Hungary). At pH = 1.0, the -NH_2_ and -NH- groups of the model compounds are protonated, while the other functional groups, i.e., -OH and -COOH are neutral. It makes the model compounds positively charged except mandelic acid (**23**), which is neutral. Although this neutral state makes mandelic acid (**23**) the most lipophilic molecule among the studied ones, it did not result in its greater distribution in the apolar phase. In contrast, it remained in the aqueous phase during the experiments. This statement is also generally valid for the studied selector molecules (**8**–**10**, **13**–**17**). Results for selectors are summarized in Table 2.

The results obtained for mandelic acid (**23**, *ee*%~0) can probably be explained by the nature of intermolecular binding forces. Coordination complexes are formed by secondary binding forces between the stereoelectronically complementary groups inside the membrane. In the case of the selector molecules, their ester groups are responsible for interacting with the guest molecules. The lone pairs of the *O* atoms can donate electrons to the electron-deficient species, especially to the positively charged ones. Thus, *H*-bonds, dipole–ionic and *π*-ionic interactions have the greatest contributions among the possible interactions in this case. However, they cannot be formed with mandelic acid (**23**). This observation also supports that instead of molecular polarity, non-covalent interactions are the key factors mediating the membrane affinity. Similarly, *π*-*π* interactions between the aromatic subunits also strongly affect the binding properties. The lack of these interactions makes aliphatic selector molecules [CA and MEN esters (**8**–**10** and **13**, **14**)] less suitable for distinguishing enantiomers of the studied aralkyl species (**19**–**23**). Extended planar aromatic units can also support the manifestation of steric repulsions within coordination complexes, thus selectors containing aromatic subunits are generally preferred for the separation of all types of guest molecules. Consequently, among the studied selector molecules, DBTA-12 (**17**) proved to be the best one for enantioenriching the selected model compounds. Other enantiopure lipophilic selector molecules containing aromatic subunits with a physical form of a viscous oil can also be promising alternatives in the future. The length of the alkyl chains did not influence the separation ability. Since no significant differences were obtained, data were reported only for selectors containing the longest alkyl chains (**10**, **14** and **17**). Based on the experience, the introduction of a hexyl chain is quite enough for providing sufficient lipophilicity.

It should also be considered for the future extension of the method that the introduced concept requires the chiral building blocks to be at least weakly soluble in an aqueous medium. Based on the preliminary studies, only cationic guest molecules can be effectively coordinated by the applied selector molecules. In the case of multiple stereogenic centers, it is expected that the configuration of the *sp^3^* center associated with the positively charged sites of the molecule will be decisive for the preference in enantiomeric recognition.

### 3.3. Validation of Enantioenrichment of Racemic Mixtures

**Figure 4:**
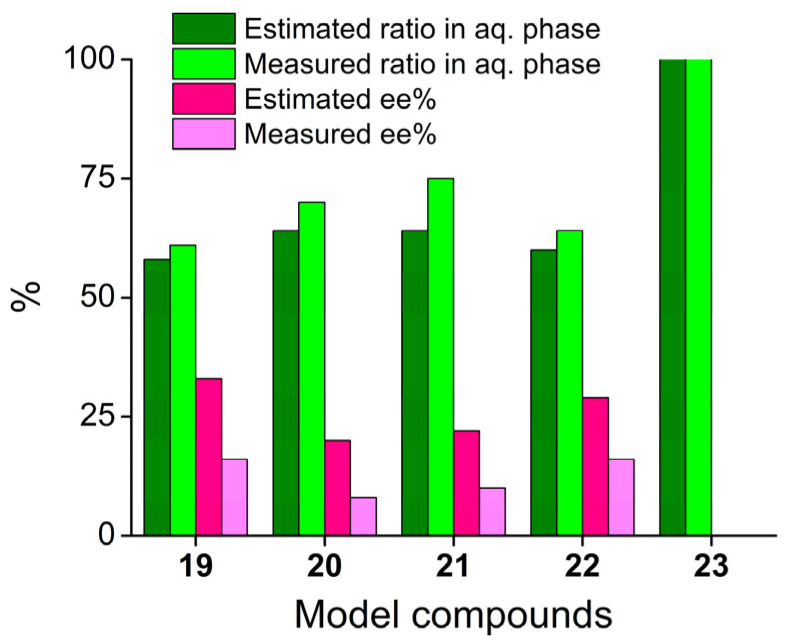
Validation of the method for enantioenrichment of racemic mixtures by using DBTA-12 (**17**) as a liquid membrane (incubation = 60 min; ratio in the aqueous phase was estimated by averaging the distributions of the isolated enantiomers obtained from studies on separability).

Since among the studied selector molecules, DBTA-12 (**17**) proved to be the best one for the enantioenrichment of the selected model compounds, it was subjected to a proof-of-concept study on applicability. Separation was carried out using the same model compounds (**19**–**23**) as racemic mixtures in the aqueous donor phase. After 1 h of incubation under the same conditions as applied for the reported preliminary studies on statistical judgment about separability, the ee% values in the aqueous phases were determined. Results are summarized in Figure 4.

The results are consistent with those of the preliminary studies on separability starting from the isolated enantiomers. The efficiency of the separations was below the estimate, which can be attributed to the competition. The presence of the other enantiomers had an opposite effect on the distribution ratios and the discrimination efficiency. In summary, significant enantiomeric enrichment could be reached in the predicted cases, which is promising for practical applicability and for the extensibility of the method in the future.

## 4. Conclusions

Herein, we report a novel concept for high-throughput enantioenrichment by the introduction of an assay platform aimed to satisfy the previously unsolved problems of resolution on a subpreparative scale. The described method was designed along the line of simple availability and technological adaptability. Thus, widely used and commercially available scaffolds were modified by simple one-step reactions and used as easily integrable liquid membranes at the bottom of previously untreated microtiter assays. Additionally, we have characterized five new potential selector molecules and investigated their enantiodiscriminating power toward various chiral compounds.

It was demonstrated that significant enantiomeric enrichment can be reached within 1 h of incubation. By repeating the separation steps, an almost 100% parallel enantioenrichment of a large number of structurally analogue chiral compounds can be achieved faster than ever before and by using fewer resource requirements. Based on the preliminary studies, 96-well sample, 12 h assay analytical performance is expected for an almost total enrichment (assuming linearity in the efficiency of subsequent enrichment steps with 96-well assays), while the amount of the starting racemates reduces by two orders of magnitude. Furthermore, the handling of the reported assays by automated workstations broadens the spectrum of automatable chemical processes with a new direction.

## Figures and Tables

**Table 1:** Separability studies on the isolated pure enantiomers of model compounds **19**–**23** using NAP-6 (**18**) as a membrane selector (incubation = 60 min).

Model System	*D_E*,*l*,*aq._*	*D_E,p,aq._*	*β_(p)_* ^1^	Significant? ^2^	Estimated ee_aq_^3^ (%)
NAP-6 & **19**	0.56	0.30	1.89 _(*R*)_	✓	22
NAP-6(M) ^4^ & **19**	0.35	0.33	0.95 _(*R*)_	✗	0
NAP-6 & **20**	0.37	0.23	1.58 _(*R*)_	✓	17
NAP-6(M) ^4^ & **20**	0.16	0.11	1.47 _(*R*)_	✗	0
NAP-6 & **21**	2.85	2.00	1.40 _(*R*)_	✓	5
NAP-6(M) ^4^ & **21**	0.52	0.52	1.00 _(*R*)_	✗	0
NAP-6 & **22**	0.64	0.47	1.36 _(1*R*,2S)_	✓	10
NAP-6(M) ^4^ & **22**	0.32	0.27	1.19 _(1*R*,2S)_	✗	0
NAP-6 & **23**	13.3	10.1	1.31 _(*R*)_	✗	0
NAP-6(M) ^4^ & **23**	0.82	0.75	0.92 _(*R*)_	✗	0

^1^*β_*(*p*)*_* refers to the selectivity toward the preferred enantiomer over the less preferred one according to the structural preference of the selector molecule. ^2^ Judging the significance of enantiomeric discrimination based on the statistical evaluation. ^3^ Calculated according to Equation (3) reported in Section 2.2. ^4^ Studies on NAP-6 (**18**) as a selector molecule in a decane-1-ol-based apolar liquid membrane.

**Table 2:** Separability studies on the isolated, single enantiomers of the model compounds **19**–**23** using model systems containing CA-12, MEN-10 or DBTA-12 selector molecules (incubation = 60 min).

Model System	*D_E*,*l*,*aq._*	*D_E,p,aq._*	*β_(p)_* ^1^	Significant? ^2^	Estimated ee_aq_^3^ (%)
CA-12 and **19**	1.17	0.89	1.3 _(*R*)_	✓	7
CA-12 and **20**	3.35	3.00	1.12 _(*R*)_	✗	0
CA-12 and **21**	99.0	32.3	3.10 *_(S_*_)_	✗	0
CA-12 and **22**	0.45	0.41	1.10 _(1*R*,2*S*)_	✗	0
CA-12 and **23**	~100	~100	1.00 _(-)_	✗	0
MEN-10 and **19**	3.35	1.94	1.72 _(*R*)_	✓	8
MEN-10 and **20**	5.25	3.17	1.66 _(*R*)_	✓	5
MEN-10 and **21**	99.0	6.14	16.1 _(*R*)_	✓	7
MEN-10 and **22**	~100	~100	1.00 _(-)_	✗	0
MEN-10 and **23**	~100	~100	1.00 _(-)_	✗	0
DBTA-12 and **19**	3.35	0.64	5.24 _(*R*)_	✓	33
DBTA-12 and **20**	3.35	1.04	3.22 *_(S_*_)_	✓	20
DBTA-12 and **21**	3.55	1.00	3.55 _(*R*)_	✓	22
DBTA-12 and **22**	3.35	0.72	4.62 _(1*S*,2*R*)_	✓	29
DBTA-12 and **23**	~100	~100	1.00_(-)_	✗	0

^1^*β_*(*p*)*_* refers to the selectivity toward the preferred enantiomer over the less preferred one according to the structural preference of the selector molecule. ^2^ Judging the significance of enantiomeric discrimination based on the statistical evaluation. ^3^ Calculated according to Equation (3) reported in Section 2.2.

## Data Availability

The authors confirm that the data supporting the findings of this study are available within the article and its Supplementary Materials.

## Supplementary Materials

The following supporting information can be downloaded at: https://www.mdpi.com/article/10.3390/membranes13010094/s1, Section S1: UV–Vis calibration curves (10^−4^–10^−5^ mol·L^−1^) for determining the concentration of the model compounds (**19**–**23**) at pH = 1.0 in aqueous solutions; Section S2: ^1^H-NMR and ^13^C-NMR spectra of the new compounds; Section S3: Preliminary studies on kinetics of separability using UV–Vis absorption spectroscopy to determine the concentration of the aqueous phases.
