# Supplementary material for: Enantiodiscriminating Lipophilic Liquid Membrane-Based Assay for High-Throughput Nanomolar Enantioenrichment of Chiral Building Blocks
Source: Membranes (Basel). 2023 Jan 11;13(1):94. doi: 10.3390/membranes13010094 (PMC9862411; doi:10.3390/membranes13010094)
Supplement: Supplementary file 1 [file membranes-13-00094-s001.zip › membranes-2137608-supplementary.pdf]

Supporting Information

Enantiodiscriminating lipophilic liquid membrane-based assay for high-throughput nanomolar enantioenrichment of chiral building blocks

Bálint Jávor [1], Panna Vezse [1], Ádám Golcs [1,*], Péter Huszthy [1] and Tünde Tóth [1,2]

[1] Department of Organic Chemistry and Technology, Budapest University of Technology and Economics, Szent Gellért tér 4, H-1111 Budapest, Hungary
[2] Centre for Energy Research, Konkoly-Thege Miklós út 29-33, H-1121 Budapest, Hungary
* Correspondence: golcs.adam@edu.bme.hu

Contents

1. UV-Vis calibration curves (10^{-4}-10^{-5} mol·L^{-1}) for determining the concentration of the model compounds (**19-23**) at pH=1.0 in aqueous solutions
2. ^{1}H-NMR and ^{13}C-NMR spectra of the new compounds
3. Preliminary studies on kinetics of separability using UV-Vis absorption spectroscopy to determine the concentration of the aqueous phases

Section S1. UV-Vis calibration curves (10^{-4}-10^{-5} mol·L^{-1}) for determining the concentration of the model compounds (19-23) at pH=1.0 in aqueous solutions

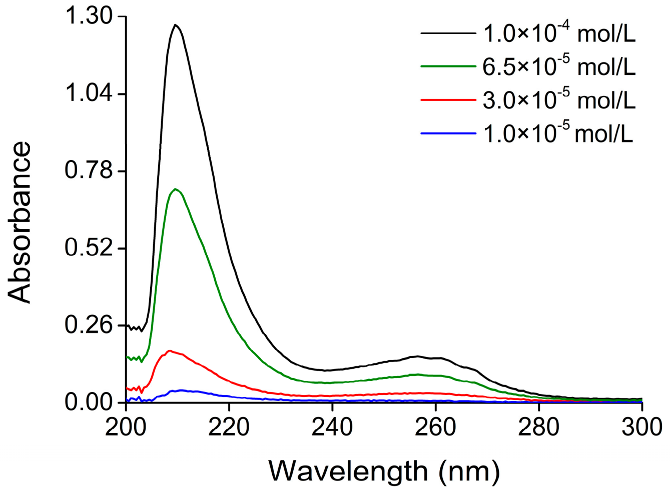

Figure S1. UV-absorption spectra of model compound **19**

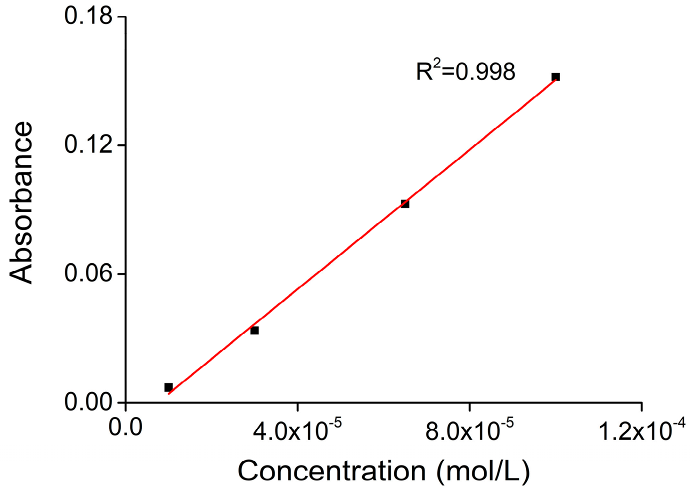

Figure S2. Calibration curve for model compound **19** at 258 nm

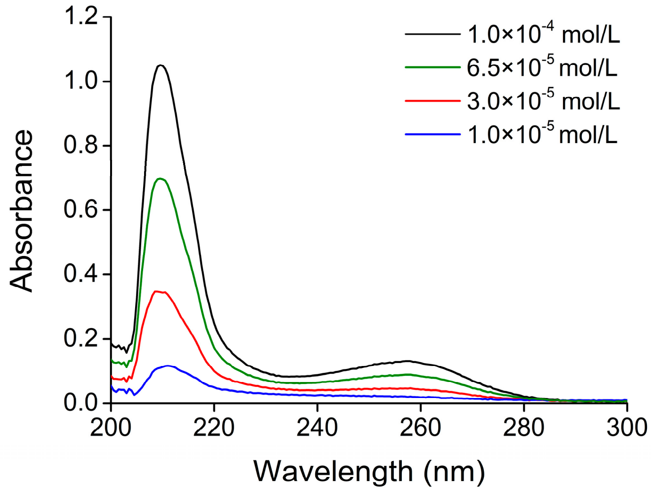

Figure S3. UV-absorption spectra of model compound **20**

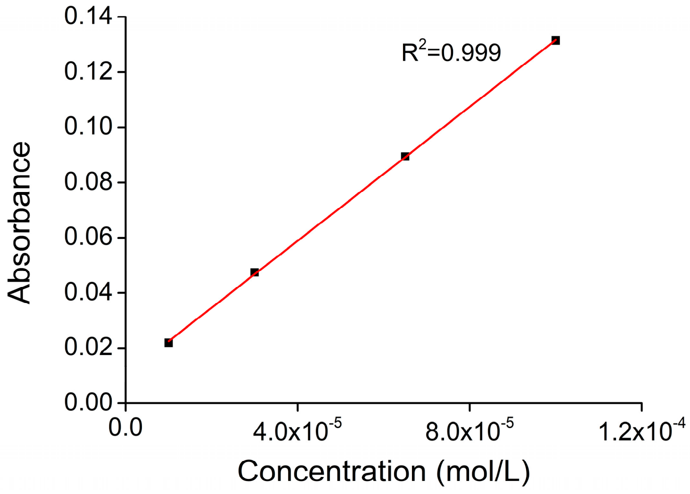

Figure S4. Calibration curve for model compound **20** at 258 nm

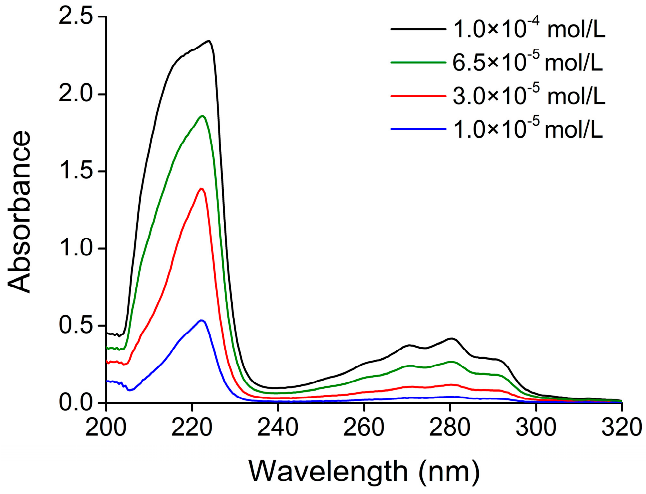

Figure S5. UV-absorption spectra of model compound **21**

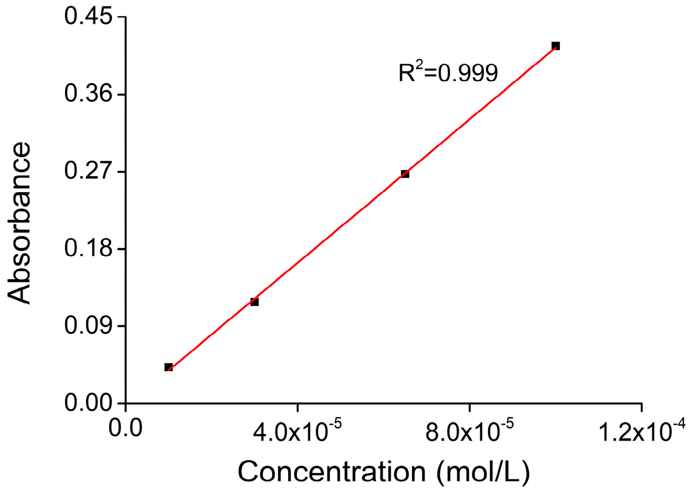

Figure S6. Calibration curve for model compound **21** at 280 nm

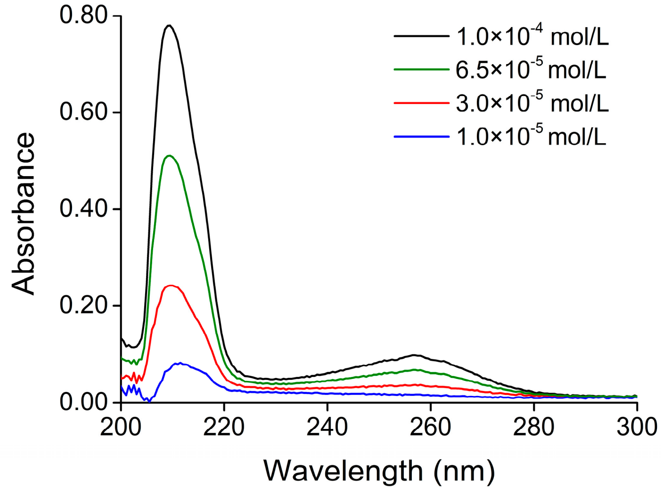

Figure S7. UV-absorption spectra of model compound **22**

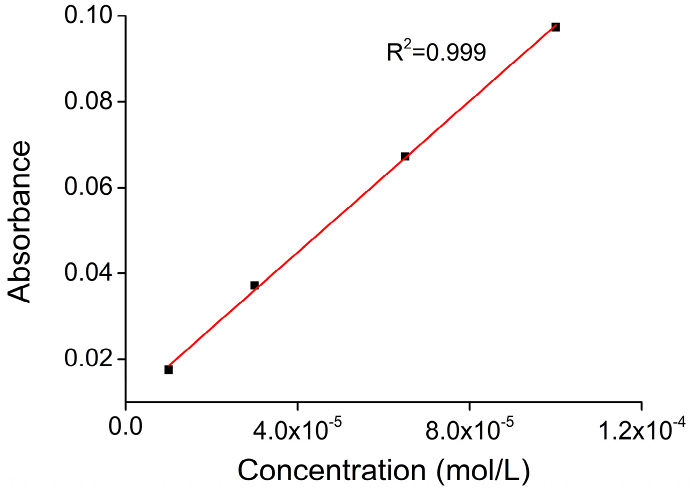

Figure S8. Calibration curve for model compound **22** at 258 nm

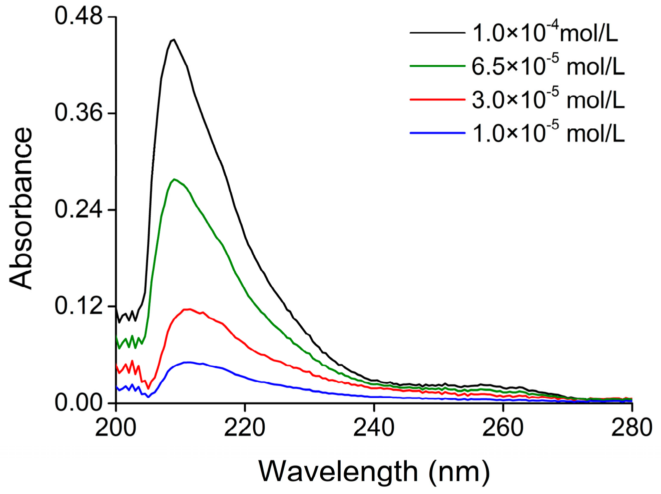

Figure S9. UV-absorption spectra of model compound **23**

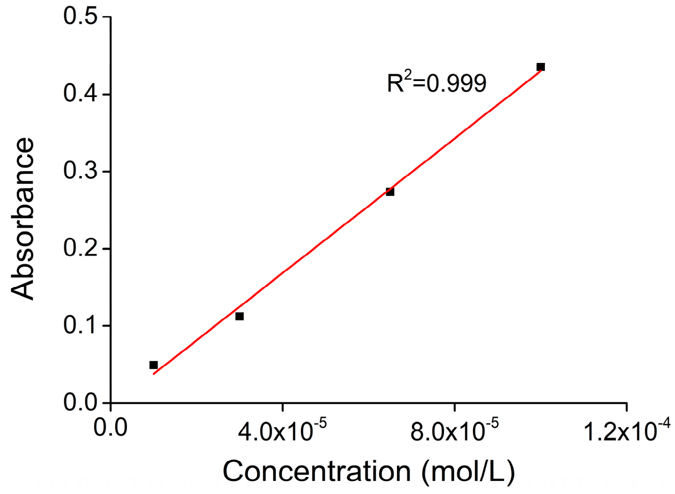

Figure S10. Calibration curve for model compound **23** at 210 nm

Section S2. ^{1}H-NMR and ^{13}C-NMR spectra of the new compounds

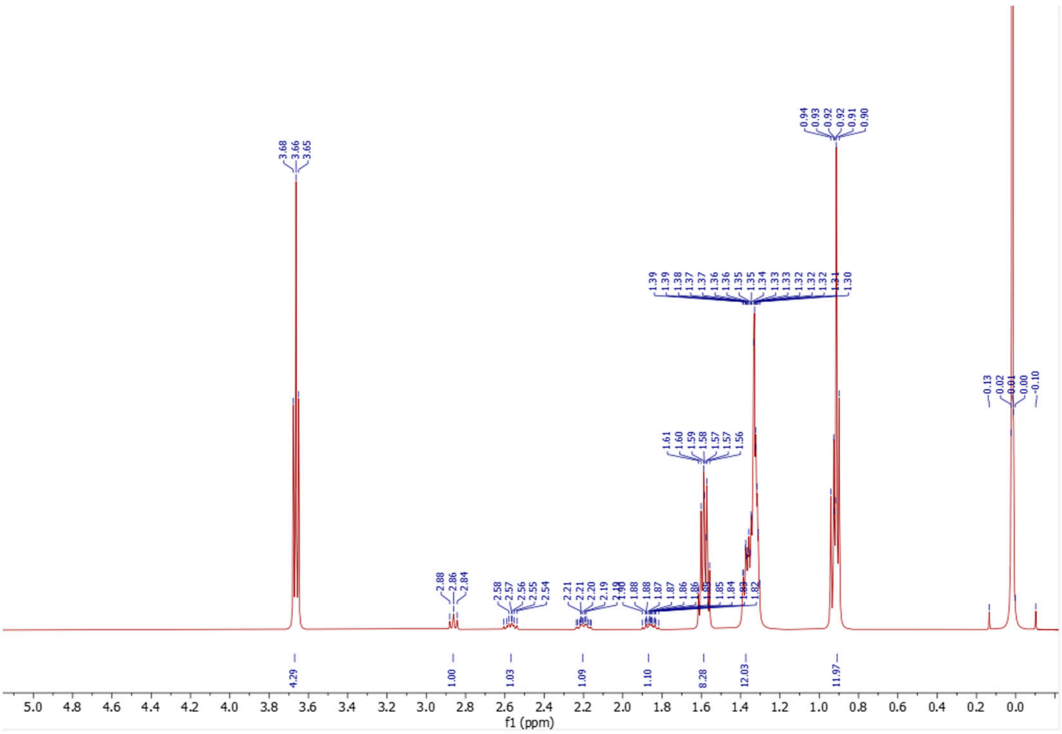

Figure S11. ^{1}H-NMR spectrum of CA-6 (**8**, solvent: $CDCl_3$)

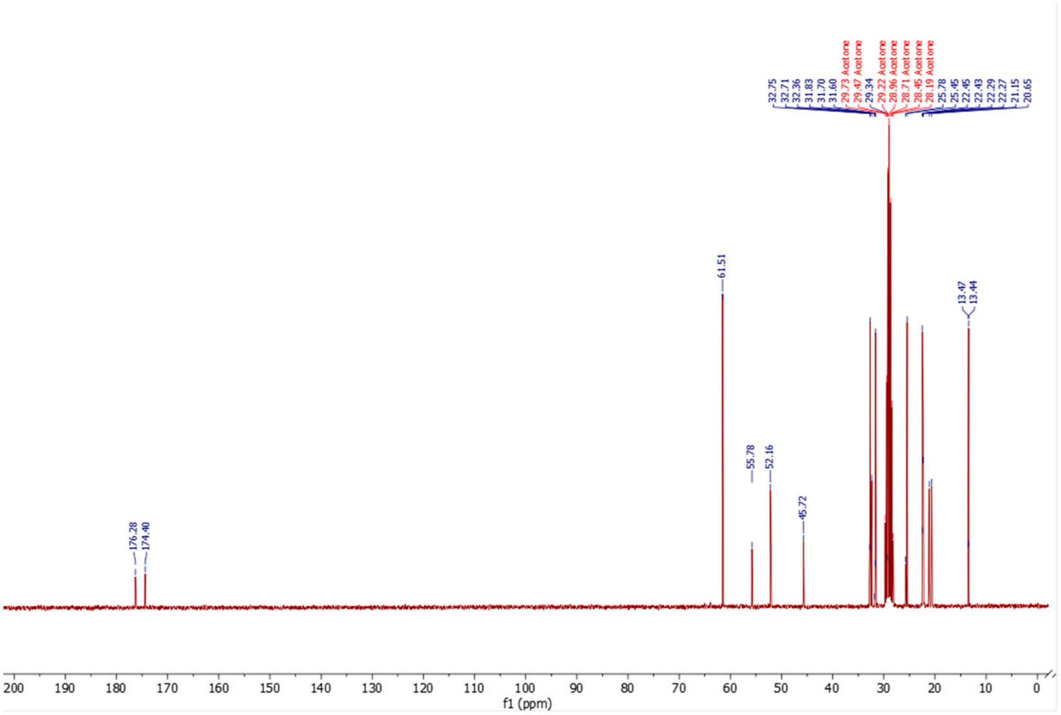

Figure S12. ^{13}C-NMR spectrum of CA-6 (**8**, solvent: acetone-d_6)

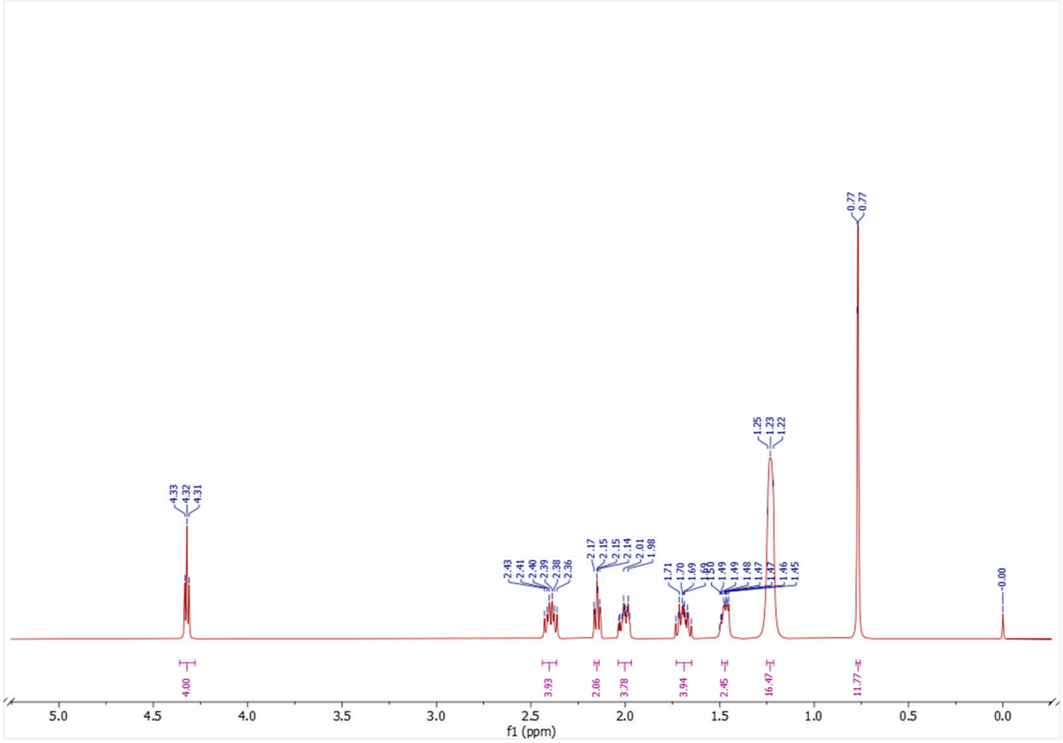

Figure S13. ^{1}H-NMR spectrum of CA-8 (**9**, solvent: acetone-d_6)

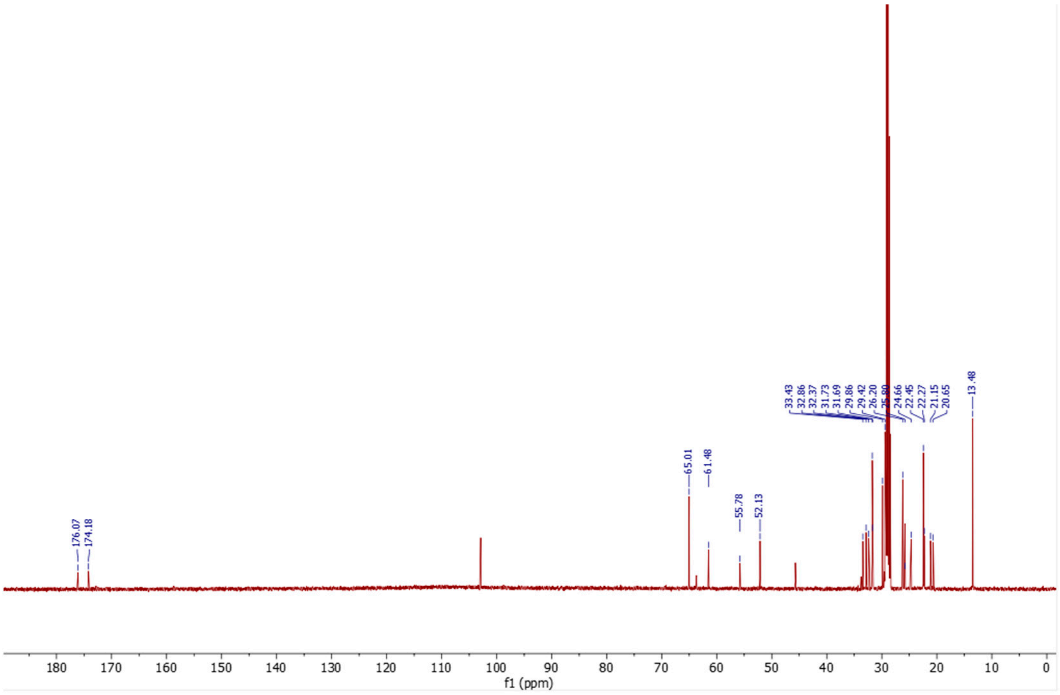

Figure S14. ^{13}C-NMR spectrum of CA-8 (**9**, solvent: acetone-d_6)

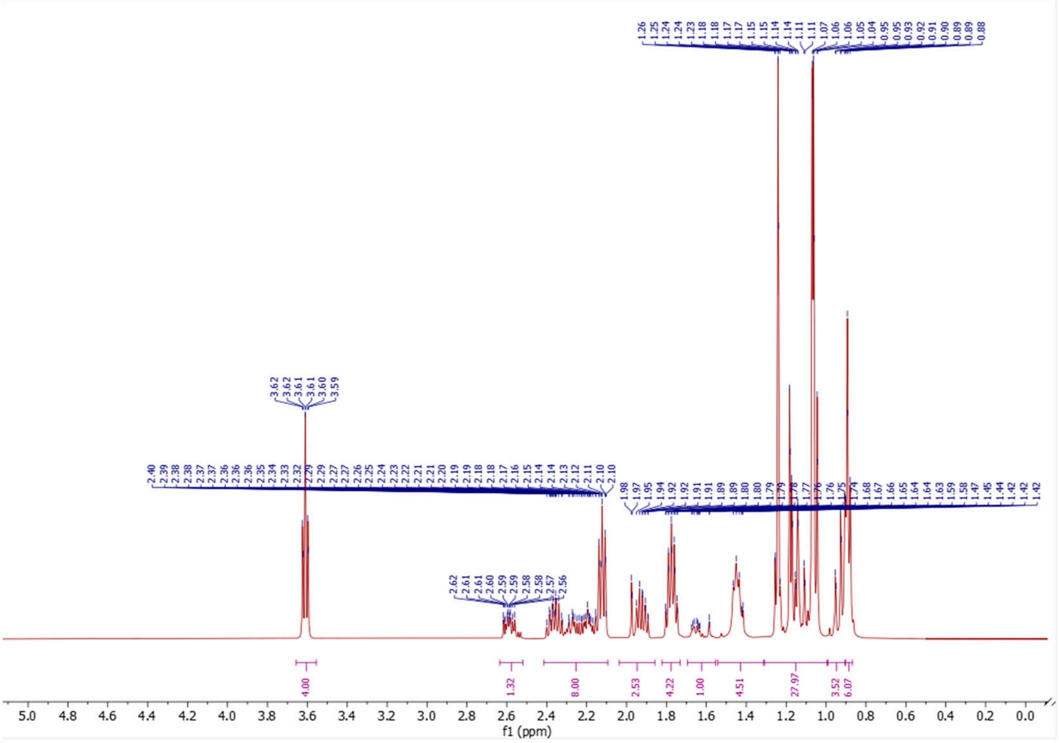

Figure S15. ^{1}H-NMR spectrum of CA-12 (**10**, solvent: $CDCl_3$)

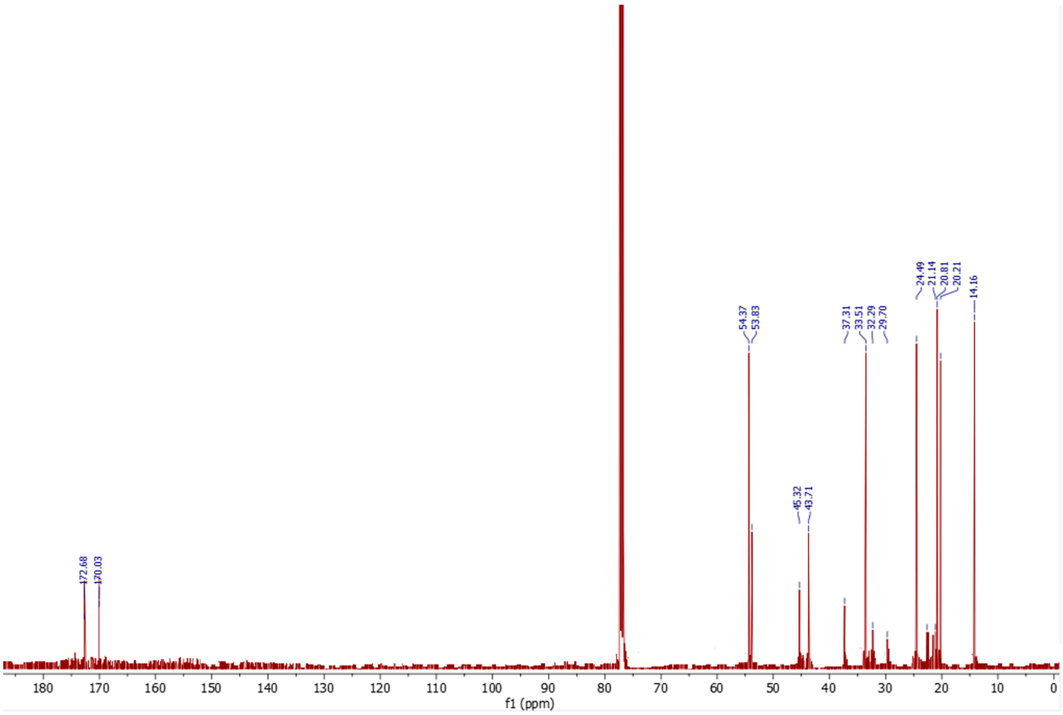

Figure S16. ^{13}C-NMR spectrum of CA-12 (**10**, solvent: $CDCl_3$)

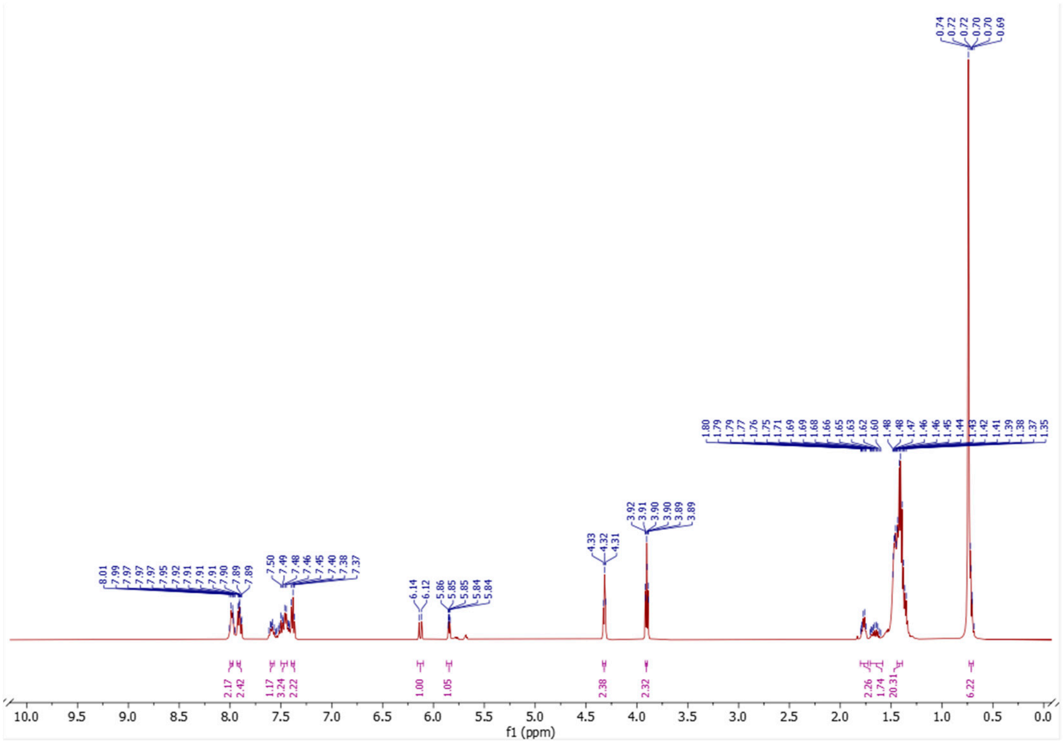

Figure S17. ^{1}H-NMR spectrum of DBTA-8 (**16**, solvent: acetone-d_6)

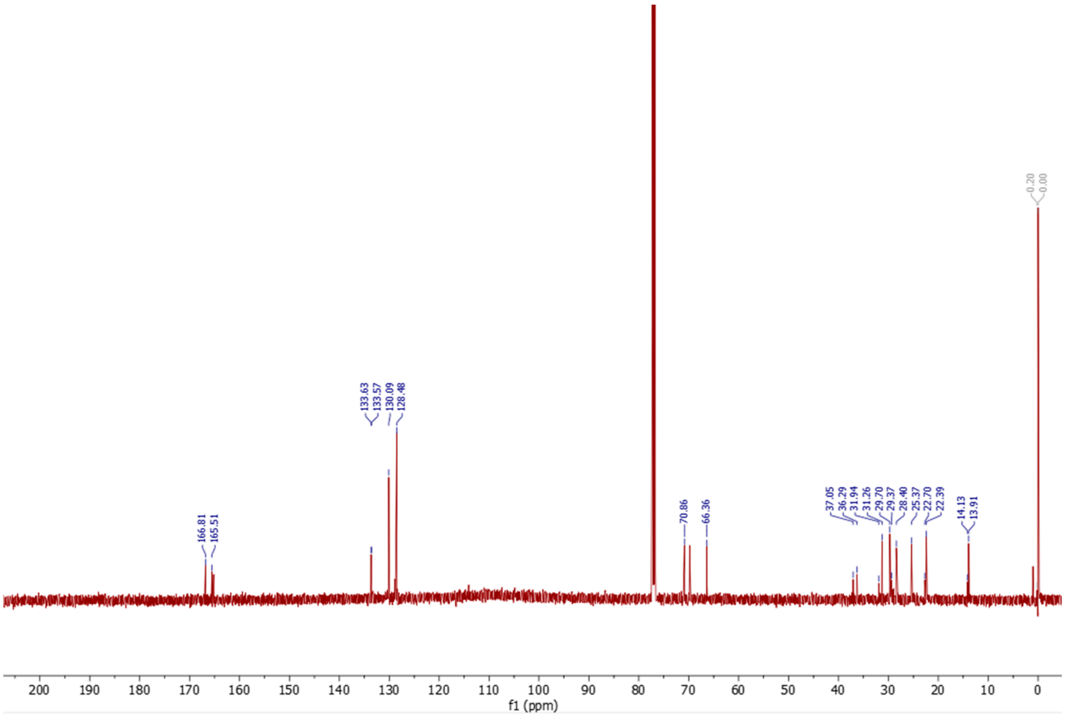

Figure S18. ^{13}C-NMR spectrum of DBTA-8 (**16**, solvent: $CDCl_3$)

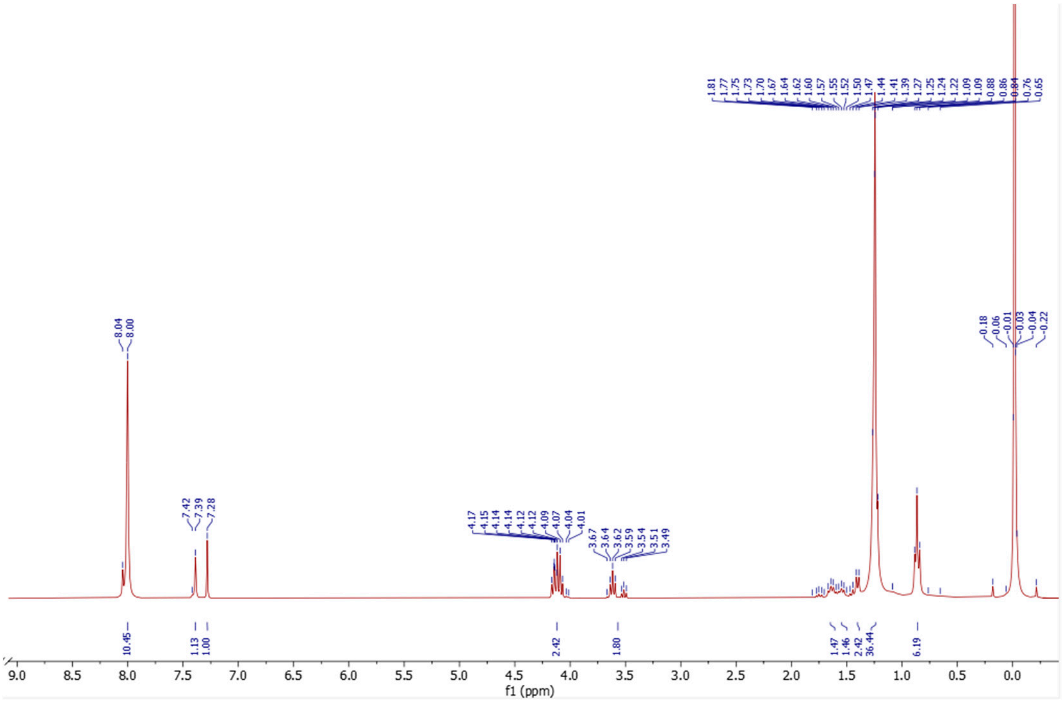

Figure S21. ^{1}H-NMR spectrum of DBTA-12 (**17**, solvent: $CDCl_3$)

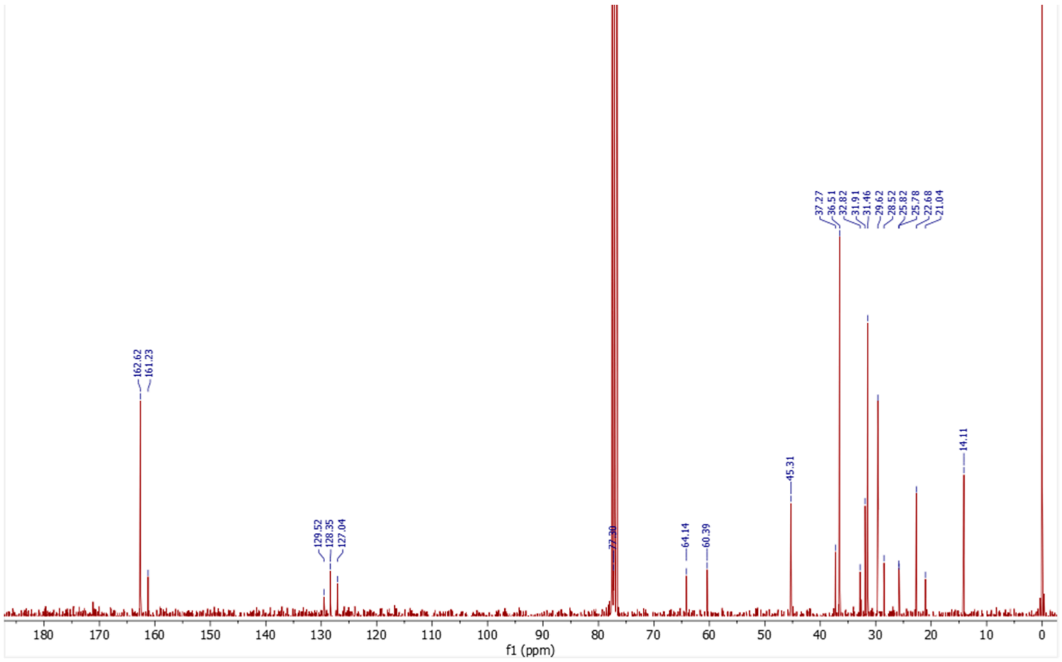

Figure S22. ^{13}C-NMR spectrum of C DBTA-12 (**17**, solvent: $CDCl_3$)

Section S3. Preliminary studies on kinetics of separability using UV-Vis absorption spectroscopy to determine the concentration of the aqueous phases

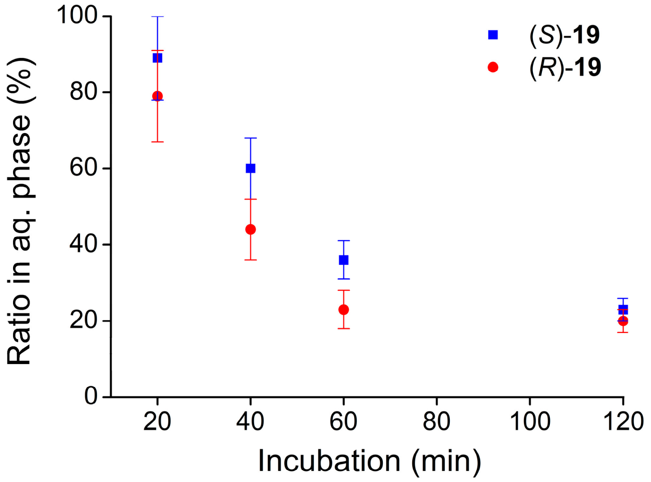

Figure S23. Studies on separability of model compound **19** using NAP-6 (**18**) as a single-component selector

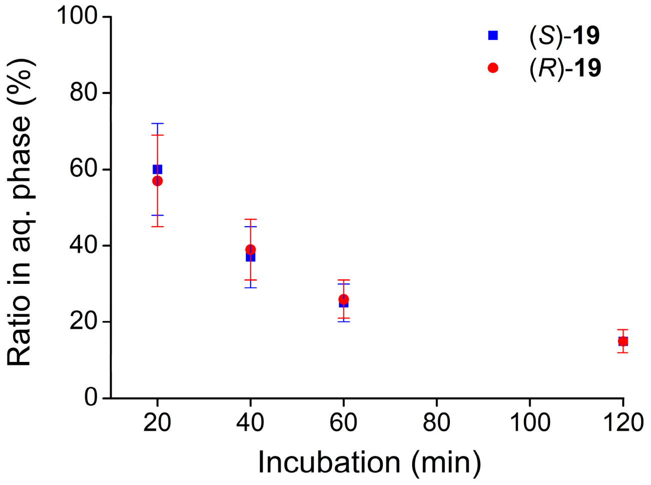

Figure S24. Studies on separability of model compound **19** using NAP-6 (**18**) as a selector molecule in a decane-1-ol based apolar liquid membrane

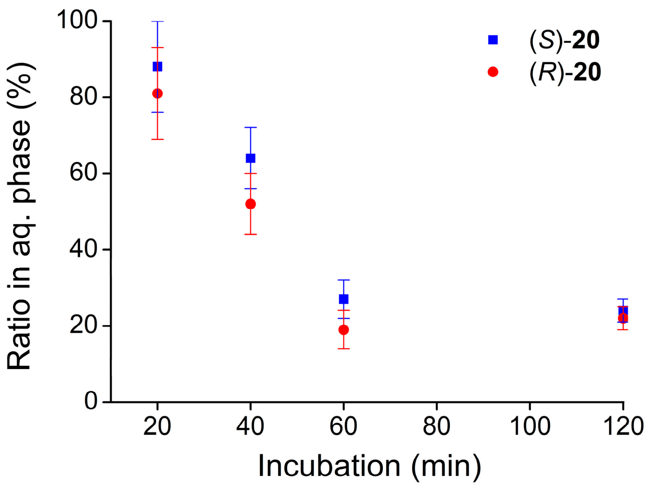

Figure S25. Studies on separability of model compound **20** using NAP-6 (**18**) as a single-component selector

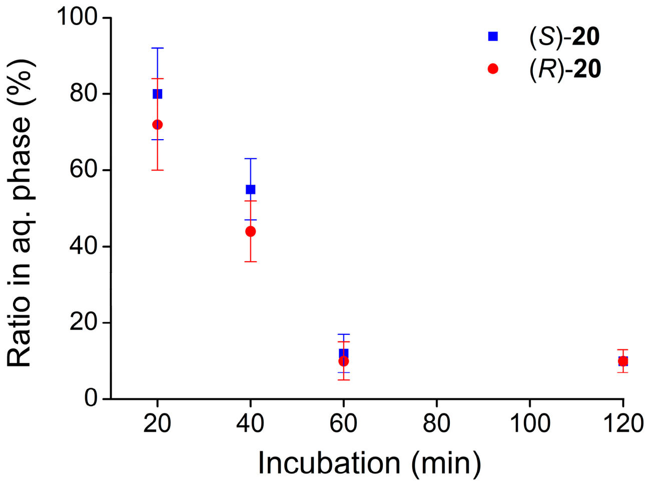

Figure S26. Studies on separability of model compound **20** using NAP-6 (**18**) as a selector molecule in a decane-1-ol based apolar liquid membrane

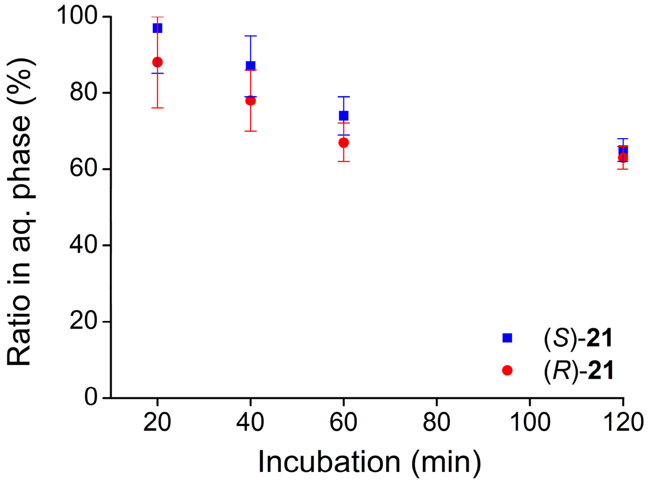

Figure S27. Studies on separability of model compound **21** using NAP-6 (**18**) as a single-component selector

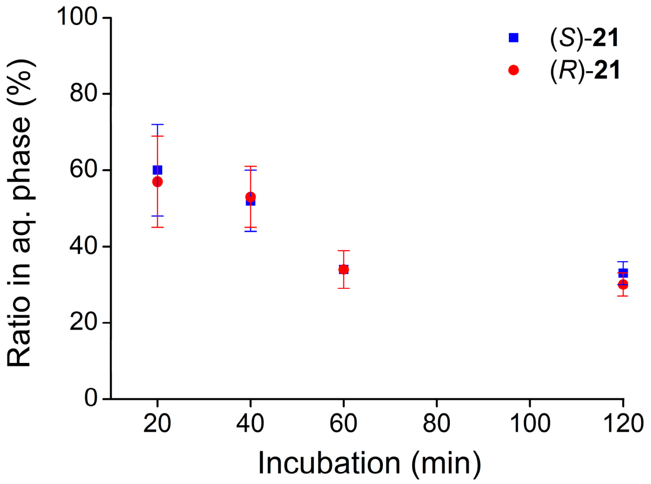

Figure S28. Studies on separability of model compound **21** using NAP-6 (**18**) as a selector molecule in a decane-1-ol based apolar liquid membrane

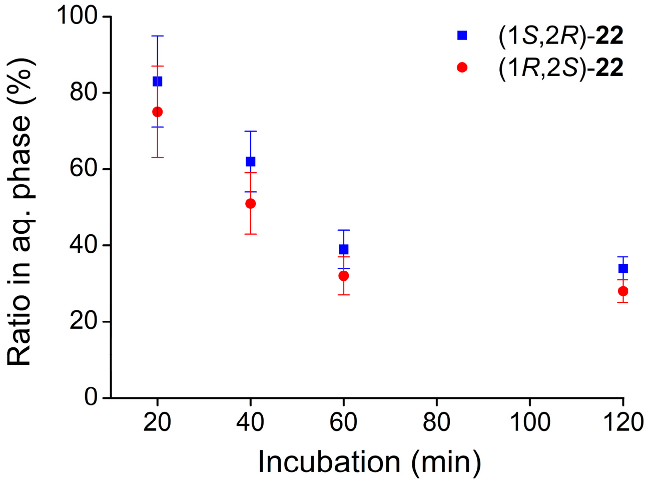

Figure S29. Studies on separability of model compound **22** using NAP-6 (**18**) as a single-component selector

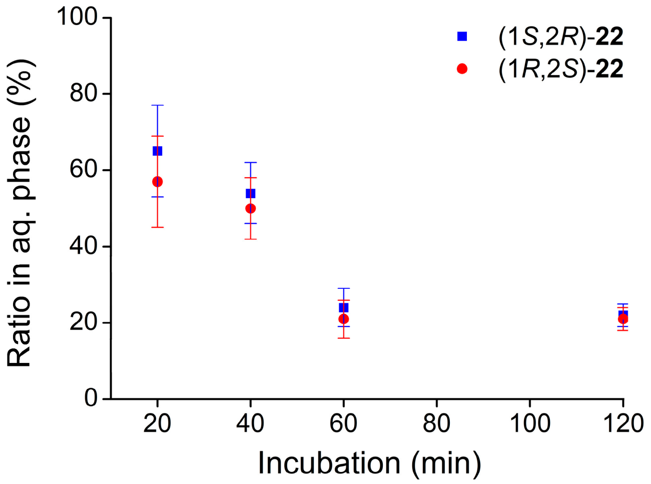

Figure S30. Studies on separability of model compound **22** using NAP-6 (**18**) as a selector molecule in a decane-1-ol based apolar liquid membrane

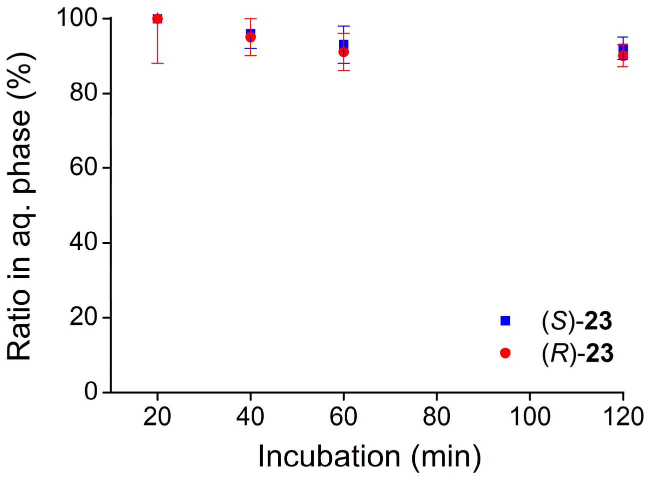

Figure S31. Studies on separability of model compound **23** using NAP-6 (**18**) as a single-component selector

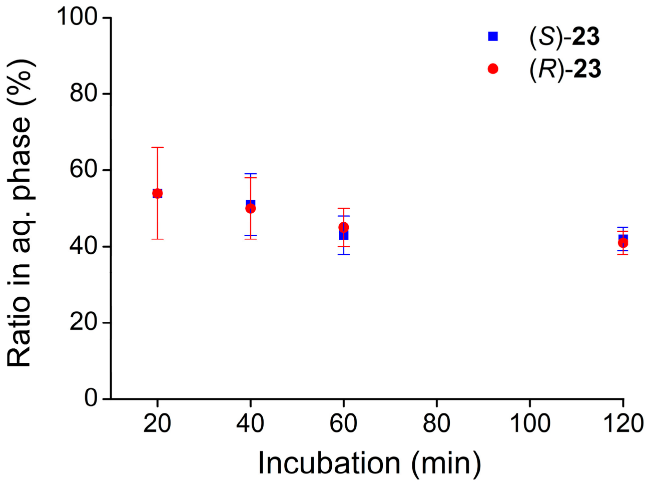

Figure S32. Studies on separability of model compound **23** using NAP-6 (**18**) as a selector molecule in a decane-1-ol based apolar liquid membrane

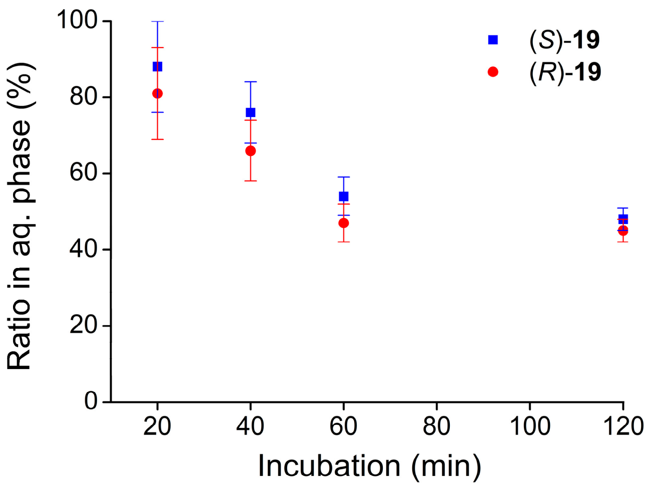

Figure S33. Studies on separability of model compound **19** using CA-12 (**10**) as a selector molecule in a decane-1-ol based apolar liquid membrane

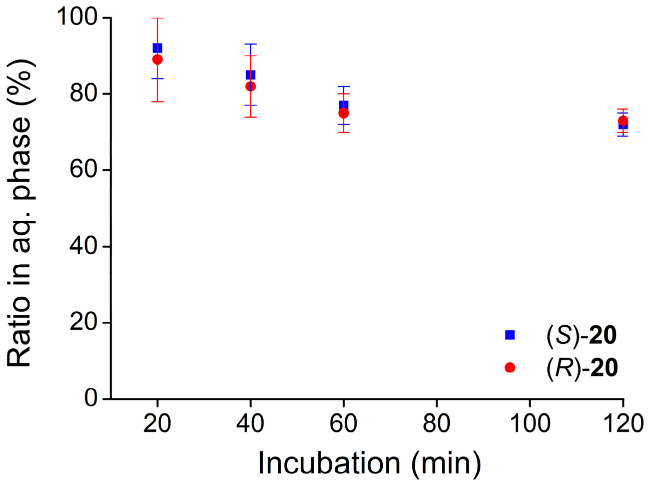

Figure S34. Studies on separability of model compound **20** using CA-12 (**10**) as a selector molecule in a decane-1-ol based apolar liquid membrane

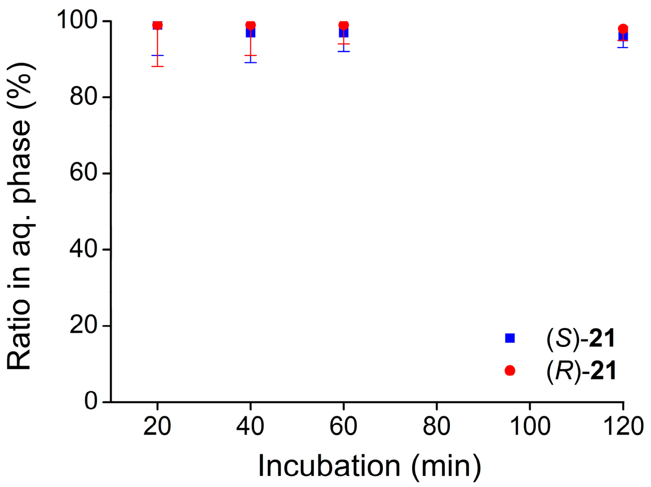

Figure S35. Studies on separability of model compound **21** using CA-12 (**10**) as a selector molecule in a decane-1-ol based apolar liquid membrane

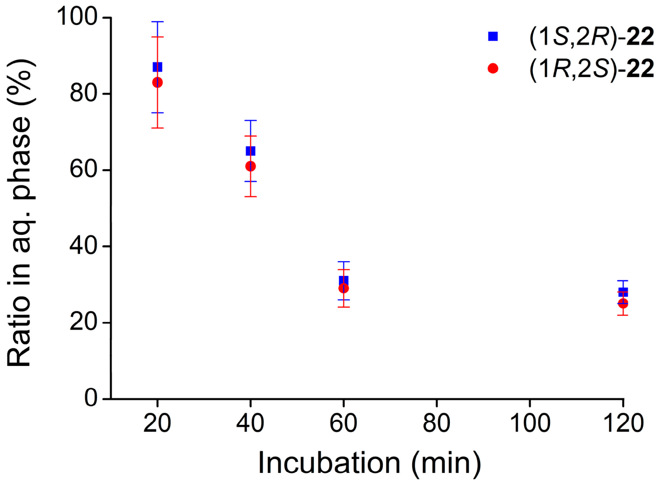

Figure S36. Studies on separability of model compound **22** using CA-12 (**10**) as a selector molecule in a decane-1-ol based apolar liquid membrane

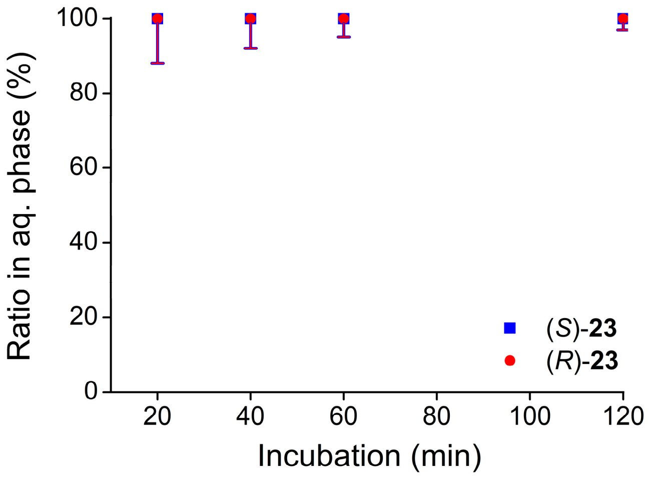

Figure S37. Studies on separability of model compound **23** using CA-12 (**10**) as a selector molecule in a decane-1-ol based apolar liquid membrane

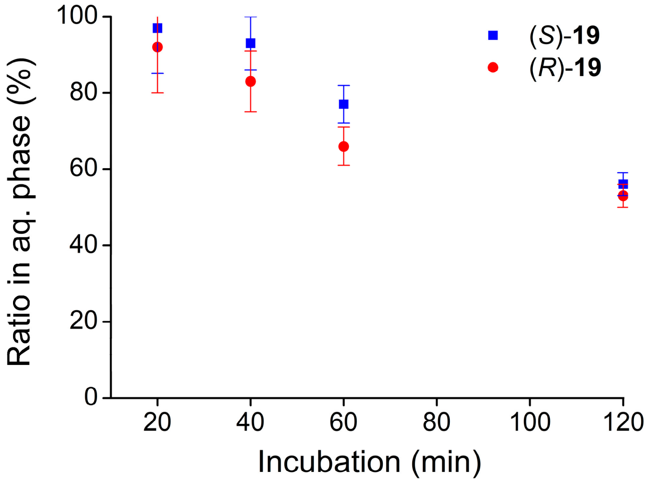

Figure S38. Studies on separability of model compound **19** using MEN-10 (**14**) as a single-component selector

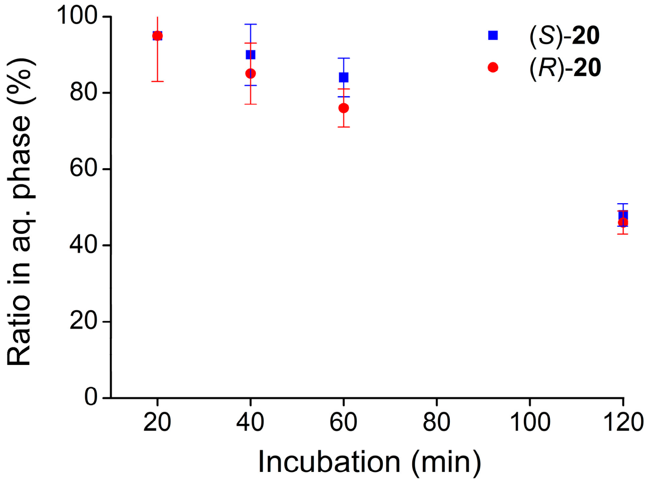

Figure S39. Studies on separability of model compound **20** using MEN-10 (**14**) as a single-component selector

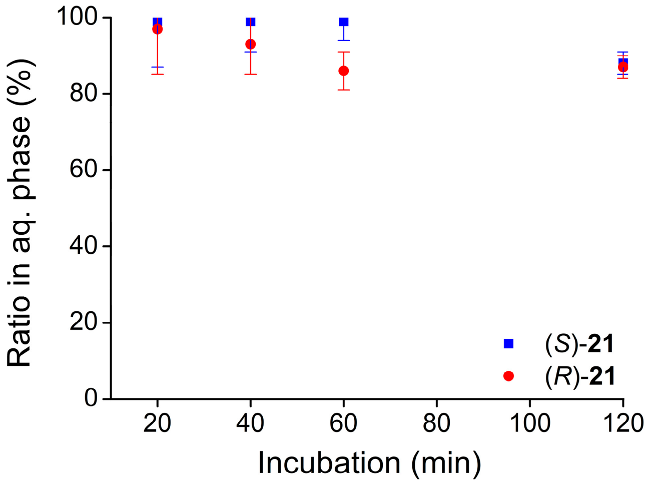

Figure S40. Studies on separability of model compound **21** using MEN-10 (**14**) as a single-component selector

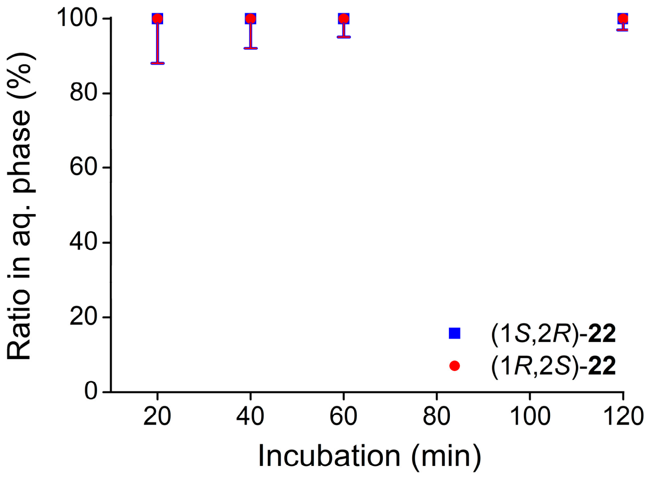

Figure S41. Studies on separability of model compound **22** using MEN-10 (**14**) as a single-component selector

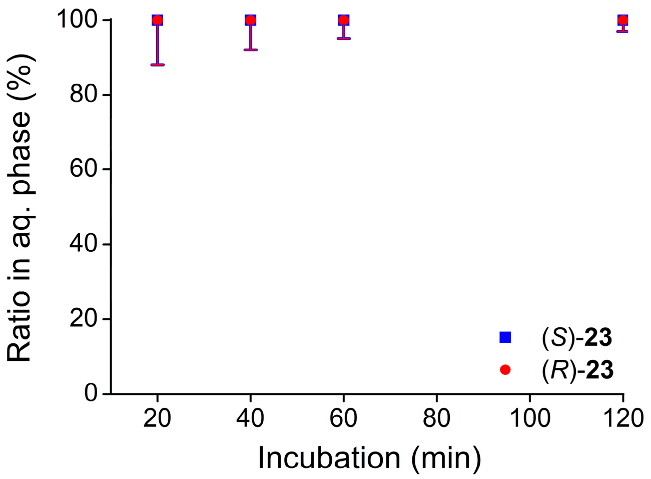

Figure S42. Studies on separability of model compound **23** using MEN-10 (**14**) as a single-component selector

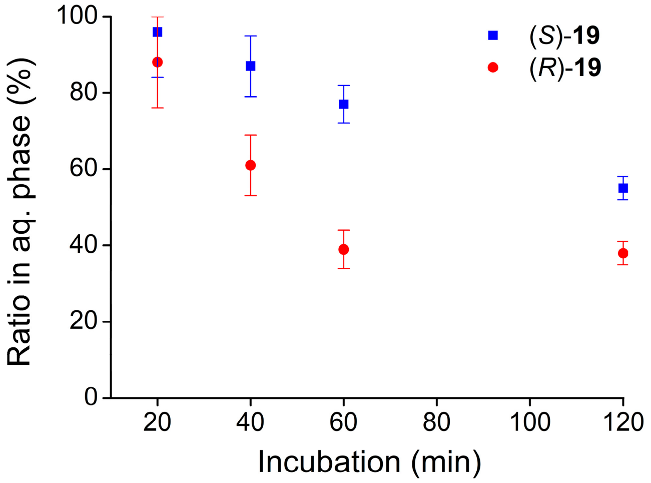

Figure S43. Studies on separability of model compound **19** using DBTA-12 (**17**) as a single-component selector

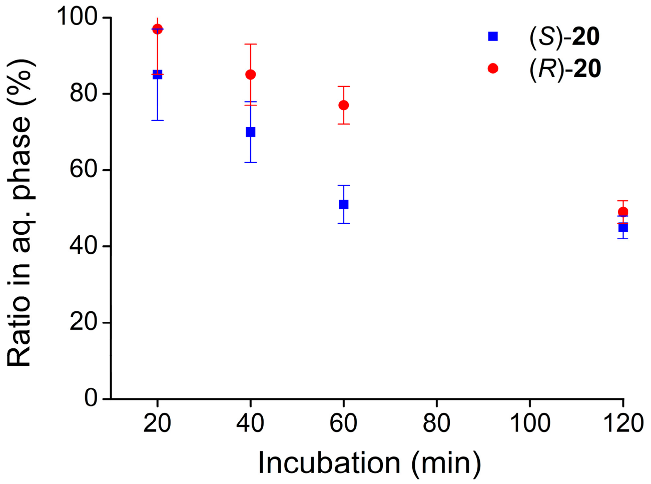

Figure S44. Studies on separability of model compound **20** using DBTA-12 (**17**) as a single-component selector

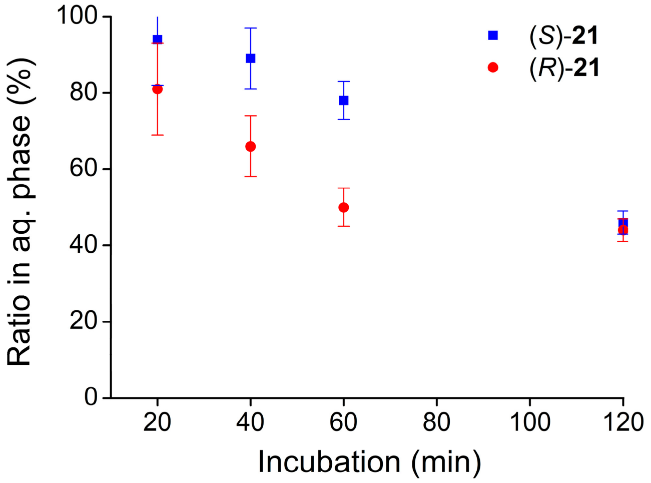

Figure S45. Studies on separability of model compound **21** using DBTA-12 (**17**) as a single-component selector

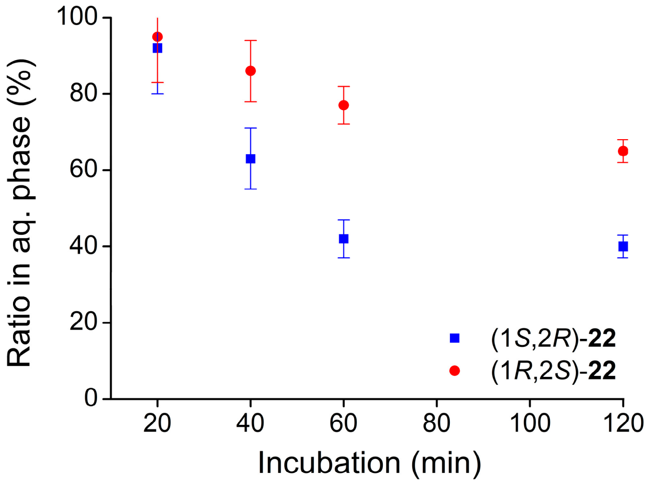

Figure S46. Studies on separability of model compound **22** using DBTA-12 (**17**) as a single-component selector

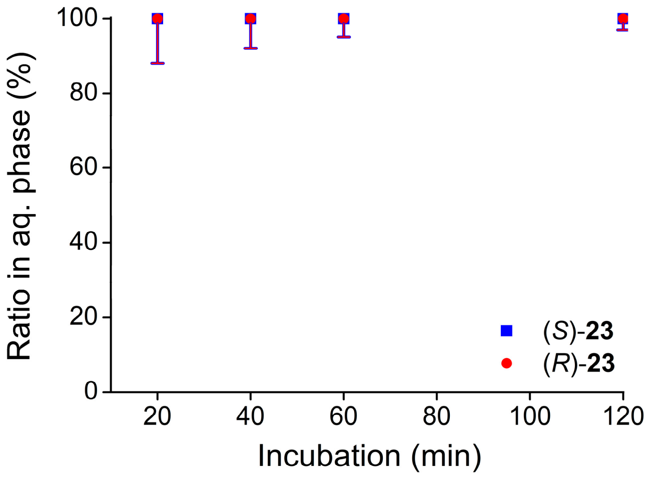

Figure S47. Studies on separability of model compound **23** using DBTA-12 (**17**) as a single-component selector
